# Layer‐by‐Layer Fabrication of Fullerene‐Intercalated Orthogonal Molecular Architectures Enhances Thermoelectric Behavior of Graphene‐Based Nanodevices

**DOI:** 10.1002/smll.202507988

**Published:** 2025-08-29

**Authors:** Ali Ismael, Xintai Wang, Bashayr Alanazi, Alaa Al‐Jobory, Colin J. Lambert

**Affiliations:** ^1^ Physics Department Lancaster University Lancaster LA1 4YB UK; ^2^ Department of Physics College of Education for Pure Science Tikrit University Tikrit Iraq; ^3^ Zhejiang Mashang GM2D Research Institute, Cangnan Wenzhou Zhejiang 325800 China; ^4^ College of Chemistry and Materials Engineering Wenzhou University Wenzhou Zhejiang 325035 China; ^5^ Department of Physics College of Science Northern Bord University Arar Saudi Arabia; ^6^ Department of Physics College of Science University of Anbar Anbar Iraq

**Keywords:** layer‐by‐layer fabrication, molecular electronics, nano‐fabrication, thermoelectric optimization

## Abstract

Despite the significant potential of molecular‐scale devices for miniaturized electronics and energy conversion applications, conventional self‐assembled monolayers (SAMs) exhibit limitations in simultaneously optimizing electrical conductivity and thermopower due to constrained electronic pathway modulation. This study demonstrates a molecular engineering strategy employing a discretely arranged conjugated molecular backbone to construct ordered cage‐like supramolecular cavities, enabling controlled intercalation of fullerene within bipyridine‐based SAMs grown on graphene‐substrates. Quartz crystal microbalance and atomic force microscopy measurements confirmed the structural integrity of the fullerene‐trapped SAMs. Notably, intercalation efficiency was significantly enhanced upon incorporation of an additional zinc tetraphenylporphyrin (ZnTPP) “cap” on top of SAMs, which prevented the loss of fullerene trapped within the cages. Electrical characterization via Eutectic Gallium‐Indium (EGaIn)‐probe measurements revealed that fullerene‐intercalated SAMs exhibited an 8.3‐fold higher normalized conductance compared to unintercalated counterparts, without reducing the Seebeck coefficient. Theoretical calculations attributed this enhancement to fullerene‐induced Fano‐resonance near the Fermi level, which amplified electron transmission. The Seebeck coefficient reached ∼60 µV K^−1^ through series interface of “slippery” pyridine‐zinc coordination and ZnTPP‐graphene π‐π coupling, while fullerene doping resulted in a similar magnitude. This cage‐like intercalation strategy proves effective for decoupling electrical conductivity and the Seebeck coefficient of SAMs, providing a robust approach for synergistic thermoelectric parameter optimization in molecular junctions.

## Graphical abstract

This study presents a cage‐like supramolecular self‐assembled monolayer on graphene that traps fullerene molecules. This intercalation, secured by a zinc tetraphenylporphyrin cap, boosts electrical conductivity 8.3‐fold without reducing the Seebeck coefficient (∼60 µV K^−1^). Theory shows Fano‐resonance enhances conduction. The strategy successfully decouples and synergistically optimizes key thermoelectric parameters in molecular junctions.

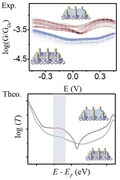

## 1 Introduction

The development of molecular‐scale devices has emerged as a frontier of nanotechnology, showing great potential in fabricating miniaturized electronic components.^[^ ^1^, ^2^, ^3^, ^4^, ^5^, ^6^ ^]^ and high‐efficiency energy conversion systems,^[^ ^7^, ^8^, ^9^, ^10^ ^]^ with precise control over charge transport at a molecular level. These range from single‐molecule junctions,^[^ ^11^, ^12^, ^13^, ^14^, ^15^ ^]^ integrating a single functional molecule into nano‐gaps, to well‐ordered molecular arrays, typically fabricated via self‐assembled monolayers.^[^ ^16^, ^17^, ^18^, ^19^, ^20^, ^21^, ^22^ ^]^ (SAMs), which are close‐packed structures, anchored to the gold substrate via an Au/S bond. Although close packing helps stability, it restricts the ability to modulate electronic pathways and phonon scattering dynamics.^[^ ^23^, ^24^, ^25^, ^26^, ^27^ ^]^ Our previous studies revealed that alternative hybrid architectures exhibit substantially enhanced Seebeck coefficients (≈60 µV K^−1^).^[^ ^28^ ^]^ compared to conventional Au‐S SAM systems (5–30 µV K^−1^).^[^ ^23^, ^29^, ^30^, ^31^, ^32^, ^33^, ^34^ ^]^ This thermoelectric enhancement originates from sharp modulations in electron transmission characteristics, induced by synergistic π‐orbital coupling and dynamic pyridine‐metal coordination bonds. While this interface‐engineering strategy successfully improves both thermopower and phonon scattering efficiency, it concurrently induces significant charge transport limitations, ultimately compromising the overall power factor (*PF = S^2^G*) through reduced electrical conductivity. In this work, we demonstrate a novel molecular engineering approach utilizing discretely arranged molecular frameworks to create ordered “*cage‐like*” supramolecular cavities. This architecture enables controlled intercalation of fullerene derivatives within the SAM matrix, establishing an optimized material platform for high‐performance thermoelectric energy conversion systems.

**Figure 1:**
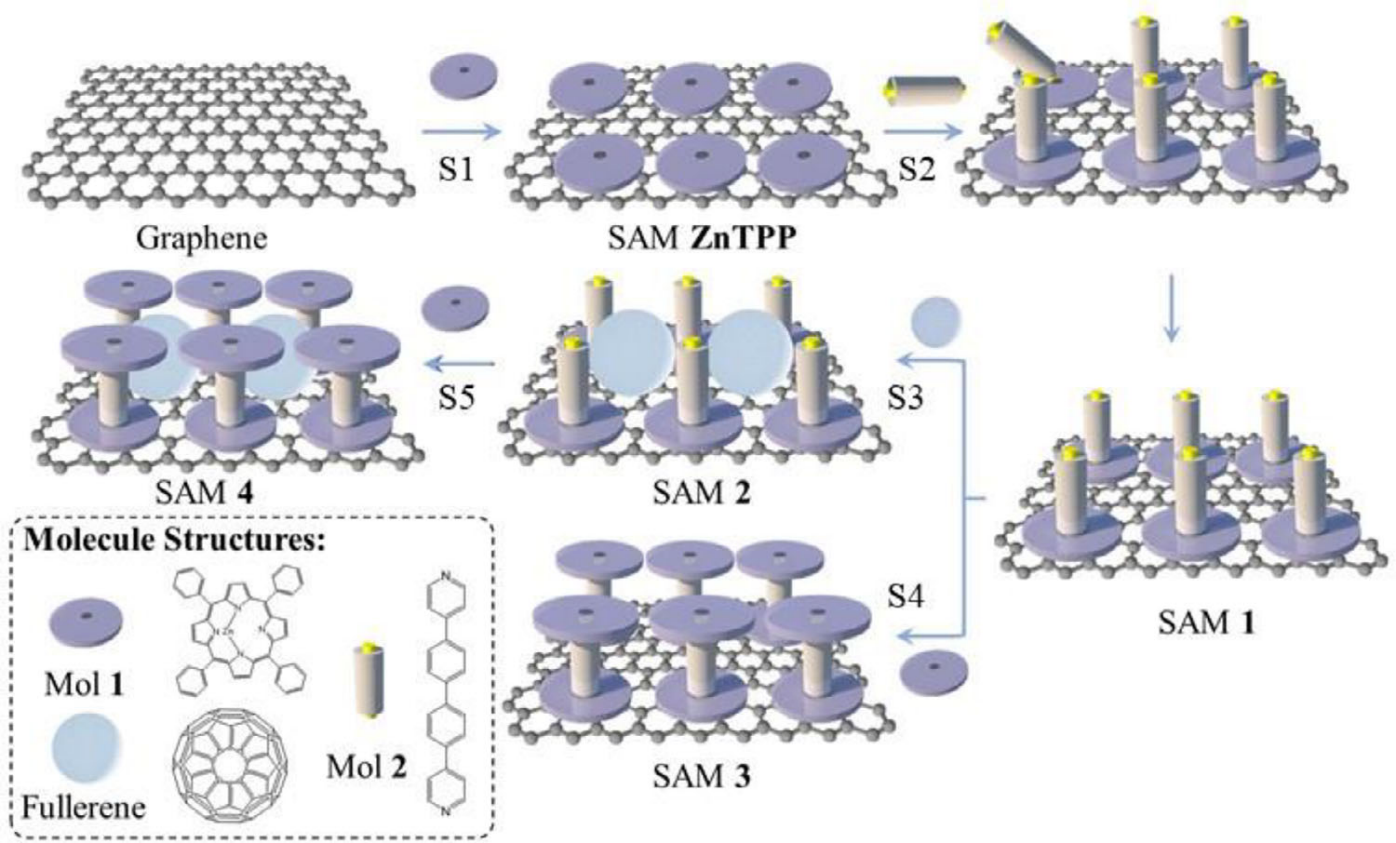
Molecules and schematic workflow of SAMs fabricated in this work. **S1**: Deposition of ZnTPP molecules onto the graphene surface via *π–π* stacking. **S2**: Growth of pyridine‐anchored molecular wires coordinated to the Zn centers of the deposited ZnTPP molecules. **S3**: Intercalation of fullerene (C_60_) molecules into the forming molecular cage structures. **S4 & S5**: Sealing of the molecular cage by coordinative coupling of a second ZnTPP molecule to the exposed pyridine termini of the molecular wires.

Building upon our previous methodology,^[^ ^28^ ^]^ we employed a layer‐by‐layer fabrication strategy to construct the SAMs on chemical‐vapor‐deposited (CVD) single‐layer graphene (SLG) substrates supported on copper. Initially, a highly ordered monolayer of zinc tetraphenylporphyrin (ZnTPP, mol **1**) was self‐assembled on SLG through *π–π* stacking interactions. Subsequently, 4,4′‐di(4‐. pyridyl)biphenyl rigid backbone molecules (mol **2**) were directionally anchored onto the Zn coordination sites through axial bonding between pyridine lone pairs and zinc centers. This molecular‐engineering approach produced vertically aligned nanostructures, with mol **2** in an orthogonal orientation relative to the plane of the ZnTPP array (**Figure** **1**, SAMs **1**). Nano‐scratching characterization confirmed this structural configuration, revealing a film thickness of ≈1.5 nm that aligns with the theoretical molecular length (1.8 nm) derived from density functional theory (DFT) calculations. Capitalizing on the inherent ≈1.6 nm inter‐metallic atom spacing, we introduced C_60_ fullerenes into the SAM matrix. The vertical alignment of mol **2** generated well‐defined cavities geometrically compatible with fullerene encapsulation. Controlled immersion of the SAM **1** in a C_60_ solution yielded SAM **2**, where fullerenes occupied the engineered cavity spaces (Figure 1, SAMs **2**). Final system integration was achieved by immersing both fullerene‐intercalated and unintercalated reference SAMs in a ZnTPP solution. The zinc is coordinated with the pyridine termini of mol **2**, forming stable host‐guest complexes (Figure 1, SAMs **4**) or maintaining empty cavities in unintercalated controls (Figure 1, SAMs **3**). Finally, a closely packed Au/S SAMs system, with oligo‐phenyl backbone (TPT), was compared with the in‐fullerene solution as a reference.

## 2 Result and Discussion

### 2.1 SAMs Characterization

**Figure 2:**
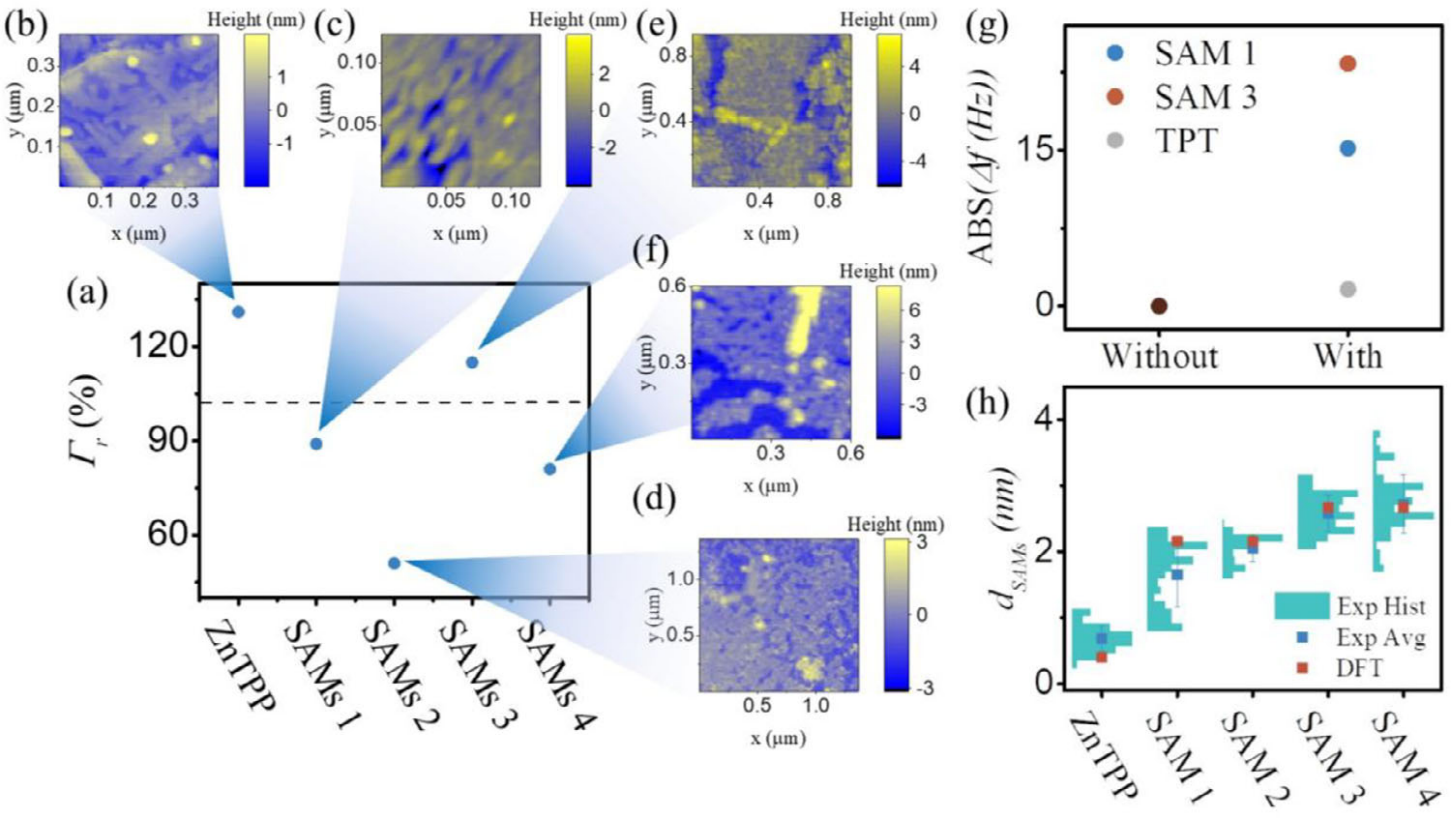
a) Surface coverage of different SAMs obtained via QCM (dash line indicate ideal coverage in theory), b–f) AFM topography image for SAM ZnTPP and **1–4**, g) shift of QCM frequency before and after fullerene intercalation for different SAMs, h) film thickness of different SAMs measured via nanoscrathing, and comparing with DFT calculated thickness in ideal growth.

Quartz crystal microbalance (QCM) measurements were employed to monitor the mass shift (*Δm_measure_*) on the substrate surface, which reflects the status of molecular layer growth, via *Γr*, representing the relative surface coverage calculated using the equation:

(1) $$\Gamma_{r}=\Delta m_{\mathrm{measure}}/\Delta m_{\mathrm{Theory}}\;\times \;100\%$$

where *Δ*m_Theory_ is the theoretically calculated mass shift for idealized, complete molecular growth according to the Figure 1 structure, with the occupation area of ZnTPP molecules set to 160 Å^2^.^[^ ^35^ ^]^ **Figure** **2**a shows the *Γr* values for the different SAMs investigated in this work. ZnTPP SAMs exhibited *Γr* value >100% (≈130%), suggesting that ZnTPP growth on graphene involves a combination of mono and bilayer formation. This outcome was anticipated because *π–π* stacking between the large aromatic rings of ZnTPP molecules can promote bilayer assembly.^[^ ^35^, ^36^, ^37^ ^]^ The result was supported by AFM (Figure 2b) and nano‐scratching measurements (Figure 2h), which indicate that the measured SAM thickness (*d_SAM_*) for ZnTPP (≈0.65 nm) is greater than the DFT‐predicted thickness for a monolayer structure (≈0.4 nm).

The *Γr* for SAM **1** is ≈90%, meaning that ≈90% of Zn adatoms on the graphene substrate were occupied by the pyridine backbone. The measured film thickness of SAM **1** is ≈1.77 nm (Figure 2c–h), comparable with the DFT‐calculated thickness (2.03 nm), and significantly higher than that of ZnTPP SAMs, further evidencing the growth of bipyridine backbones in an orthogonal architecture. Although ZnTPP serves as the foundation for pyridine backbone assembly, analysis of the correlation between ZnTPP deposition extent and backbone growth reveals no strong trend (Figure S1, Supporting Information SAMs **1**). *Γ_r_* (SAMs **1**) remains nearly constant despite higher measured *Γ_r_* (SAMs **ZnTPP**), indicating that molecular backbone adsorption is not substantially affected by ZnTPP multilayer formation. This observation stems from the pyridine ligands coupling selectively with Zn atoms in the topmost ZnTPP layer; consequently, formation of additional underlying layers does not incorporated in the molecular backbone adsorption. SAM **2** exhibited a distinct shift in QCM frequency (*Δf*) relative to SAM **1** (Figure 2g), indicating the growth of fullerene on the substrate. However, *d_SAM_* for SAM **1** and SAM **2** are almost identical (Figure 2d–h), suggesting that the fullerene molecules are positioned within the cage formed by the molecular backbone rather than atop SAM **1**. Notably, the *Γr* for SAM **2** is relatively low (≈55%, Figure 2a), implying that fullerene occupied only about half of the available growth sites. This observation likely stems from the localization of fullerene via *π–π* interaction with the molecular backbones; as *π–π* stacking, being a van der Waals force, is not very strong, some fullerene molecules were removed during rinsing. SAM **3** showed an increase in *d_SAM_* relative to SAM **1** (Figure 2e–h), indicating that coupling between the ZnTPP “cap” and the pyridine termini of SAM **1** resulted in an additional layer of ZnTPP on top of SAM **1**. *Γr* > 100% indicates multilayer growth of ZnTPP attributable to *π–π* stacking. Analogous to the comparison between SAM **1** and SAM **2**, SAM **4** demonstrated comparable thickness but a distinct frequency shift relative to SAM **3** (Figure 2f–h), indicating fullerene immobilization within the molecular “cages”. Interestingly, whereas the *Γr* for SAM **2** was relatively low (≈55%), the *Γr* for SAM **4** is significantly higher (≈85%, Figure 2a). This improvement may be attributed to the fact that the additional ZnTPP cap sealed the cage, thus preventing fullerene loss during rinsing. Notably, although multilayer growth of ZnTPP could theoretically influence cage uniformity and thus decrease intercalation efficiency, Figure S1(Supporting Information) (SAMs **2** and **4**) shows no clear correlation between *Γ_r_* (SAMs **ZnTPP**) and *Γ_r_* (SAMs **2** and **4**). This is due to the remarkably smaller value for ZnTPP layer thickness (≈0.4 nm) compared to molecular backbone length (≈1.6 nm) minimizes the impact from ZnTPP bilayer formation to molecular cage quality and fullerene intercalation efficiency. For TPT SAMs, the *Δf* before and after immersion in the fullerene solution was almost negligible, revealing the challenge of achieving doping within a closely packed monolayer (Figure 2g).

### 2.2 SAMs Electric/Thermoelectric Behavior

A custom‐built Eutectic Gallium‐Indium (EGaIn)‐probe setup,^[^ ^19^, ^22^, ^23^, ^28^, ^31^ ^]^ described in previous publications, was used for electrical and thermoelectric measurements of the fabricated SAMs. Briefly, an EGaIn tip (coated with 0.7 nm native oxide layer) from a syringe served as the top electrode, making contact with the SAMs using a micromanipulator for precise contact control. A microscope facilitated estimation of the contact area between the EGaIn tip and the SAMs. For electrical characterization, a bias voltage was applied between the copper substrate and the EGaIn probe. A current preamplifier converted the measured current to voltage, which was recorded by a data acquisition card. Differential conductances (*G*) were extracted at various bias voltages from the current‐voltage (*I‐V*) curves using the relation *G = dI/dV*. The EGaIn electrode spontaneously forms a native GaOx layer (≈0.7 nm thick) in air. This enables it to maintain a stable, non‐flowing structure through a microscale mechanical framework effect, facilitating contact with molecular layers to form vertical junctions. Simultaneously, the oxide layer suppresses the diffusion of metal atoms, preventing EGaIn from damaging the molecular monolayer. However, due to the wide bandgap of GaOx, it introduces an additional energy barrier within molecular junctions, which significantly reduces the electron tunnelling probability of molecular junctions. Furthermore, as most molecules at the EGaIn‐molecule interface interact via van der Waals forces rather than direct electronic contact, conductivity measurements using EGaIn typically yield values several orders of magnitude lower than theoretical calculations. This significant discrepancy results in limited quantitative comparability between experimental and computational results. Due to this reason, we focused on the relative conductance (*G_r_*) to exclude the contact area and native oxide layer effect, thus achieving a more direct comparison with theory.^[^ ^38^, ^39^ ^]^ Due to this reason, we focused on the relative conductance (*G_r_*) to exclude the contact area and native oxide layer effect, thus achieve a more direct comparison with theory. The relative conductance is defined as the ratio of the differential conductance for the Cu/Gr/SAMs/EGaIn junction to that of the Cu/Gr/EGaIn junction, *G_r_ = G_junction_/G_graphene_*. The corresponding IV curves for all measured systems are shown in Figure S1 (Supporting Information), and the absolute measured G and power factor value (per cm^2^) were listed in Table S1 (Supporting Information).

**Figure 3:**
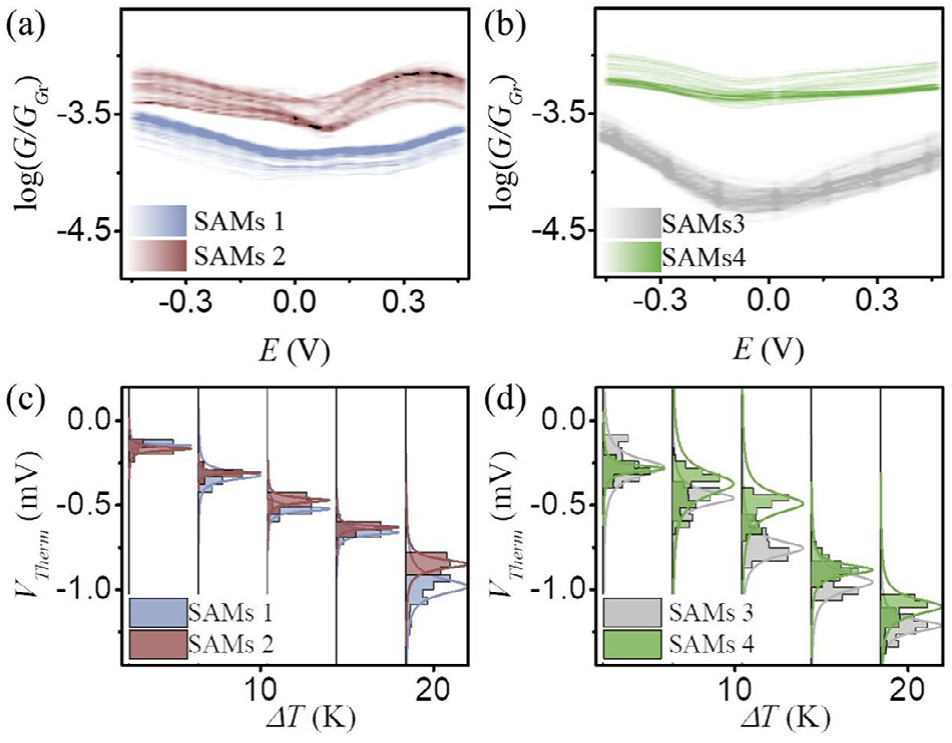
a,b) Electrical conductivity versus bias voltage and c,d) thermo‐voltage versus temperature gradient for different SAMs investigated in this work.

**Figure 4:**
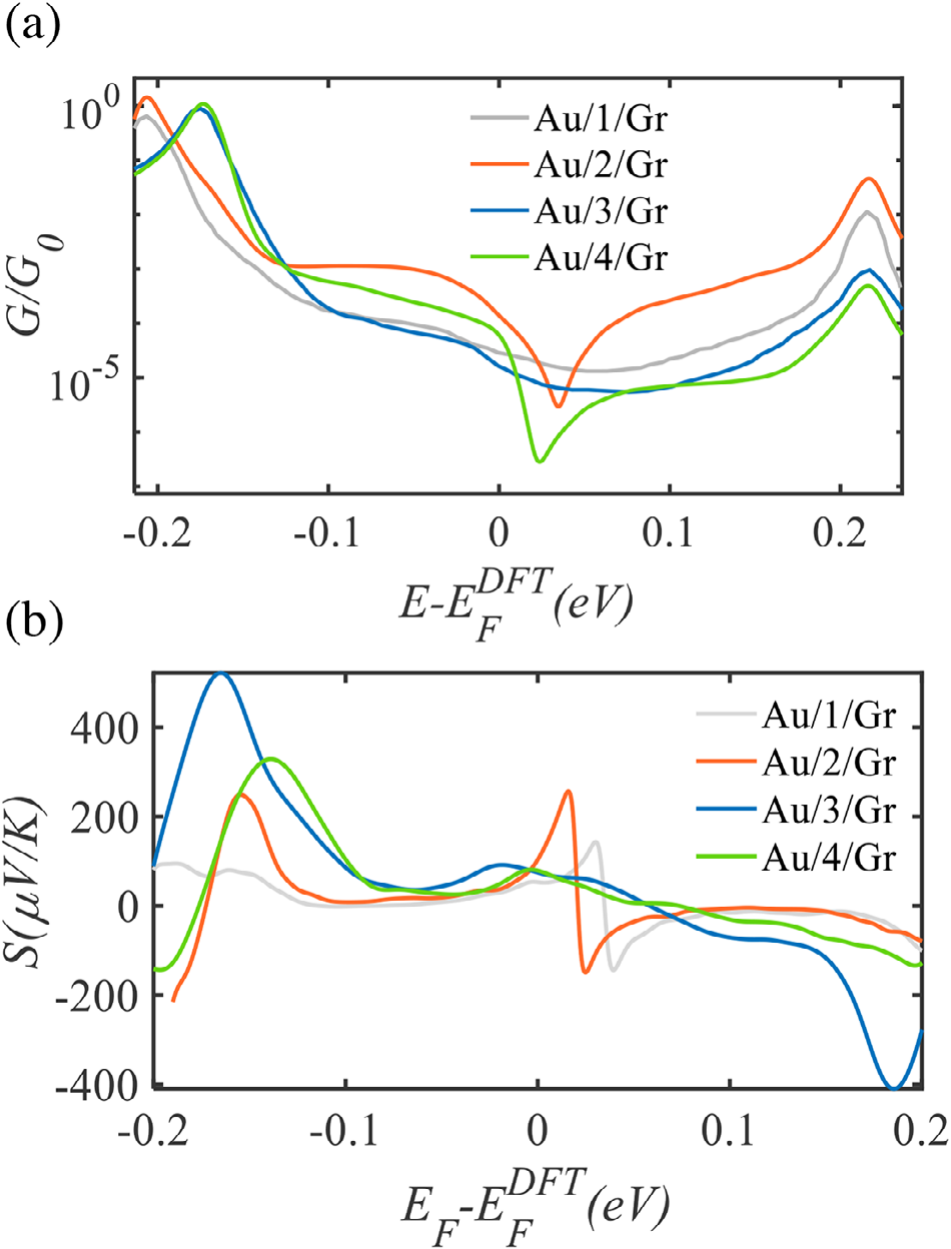
a) Calculated conductance as a function of the Fermi energy for Au/**1‐4**/Gr junctions. b) Seebeck coefficient *S* as a function of Fermi energy of the studied junctions. Seebeck coefficients of multilayers **1–4**, grey, orange, blue, and green curves, respectively.

**Figure** **3**a,b show the statistical *G_r_* curves versus bias voltage for SAMs **1–4**. The averaged *G_r_* at zero bias is listed in **Table** **1**. Each plotted curve represents statistics from at least 120 individual measurements collected from over 10 distinct sample locations. The measured G_r_ for the SAM **1** junction was 8.4 × 10^−5^, meaning comparing to the EGaIn/graphene junction, insertion of the SAM layer reduced conductivity by ≈10^4^‐fold. This substantial decrease originates from electron scattering at multiple tunneling barriers, including the ZnTPP/graphene interface, the pyridine/zinc interface, the molecular backbone itself, and the pyridine‐EGaIn interface. The *G_r_* for SAM **2** was 2 × 10^−4^, ≈2.4 times higher than that for SAM **1**. Given that SAM **1** and SAM **2** possess similar tunneling distances and interfacial contacts, this conductivity enhancement is attributed to the fullerene molecules trapped within the SAM structure. Theoretical analysis further revealed that fullerene doping does not significantly alter the relative position between the electrode Fermi level and molecular frontier orbitals (Fermi pinning effect), but markedly modulates the electron transmission spectrum, particularly inducing an anti‐resonance near the zero‐bias region (**Figure** **4**). This modulation is consistent with changes in the molecular electronic structure induced by interaction with the fullerene, which acts as an electron donor, thereby increasing the transmission probability through the SAM system and resulting in higher conductivity. SAM **3** exhibited lower conductivity than SAM **1** (*G_r_ ≈* 3.6 × 10^−5^), as expected because the additional pyridine‐Zn interface introduced an extra electron scattering barrier. Analogous to the comparison between SAMs **1** and **2**, SAM **4** also showed enhanced conductivity compared to SAM **3**, attributed to the same mechanism of fullerene‐induced conductivity enhancement. The magnitude of the conductivity enhancement from fullerene doping was quantified by the ratio *R = G_fullerene intercalate_/G_no fullerene intercalate_*. Notably, theory predicted that *R* for SAM **1** and **2** is ≈10, while in real measurement of this ratio is only ≈2.4, which is expected to arise from the loss of fullerene molecules during rinsing. This explanation is consistent with QCM data (Figure 2a), which indicated that only about half of the available sites in SAM **2** were occupied by fullerenes, leaving a portion of the molecular backbone unaffected by doping. The interpretation further supported by comparing the R value for SAMs **3** and **4**, which shown comparable R values from theory (≈8.3) and experiment (≈11), because the additional ZnTPP “cap” prevents the loss of fullerenes during rinsing, enabling fullerene intercalated in nearly all molecular cages, as implied by the higher *Γr* (≈85%, Figure 2a). While the G value at zero bias gives the intrinsic electrical conductivity of the molecular junction, the GV curve of the SAMs reveals the full map of quantum transport under energy modulation. The *G–V* curve for SAM **1** exhibits generally symmetric transport behavior, with conductance (G) remaining nearly constant between −0.3 and 0.3 V. This indicates similar transport characteristics under both positive and negative bias, consistent with DFT calculations showing the electrode Fermi level aligned mid‐gap between the HOMO and LUMO orbitals. In contrast, upon fullerene intercalation into SAM **1** (forming SAM **2**), the symmetry is broken. The conductance remains flat in the negative bias region and at low positive bias; however, a distinct transition occurs at ≈+0.15 V. Beyond this voltage, the conductance of SAM **2** increases rapidly. This behavior indicates that fullerene, acting as a strong electron acceptor, introduces an additional Fano resonance channel. This channel simultaneously enhances electron transmission efficiency (leading to higher measured conductance) and disrupts the symmetric transport behavior, as further confirmed by our DFT calculations (Figure 4). Interestingly, after the bias surpasses +0.3 V, the *G–V* curve trend shifts from increasing to decreasing. This suggests that the phases of the electron waves traversing all channels result in constructive interference ≈+0.3 V, maximizing conductance. However, as the electrode Fermi level shifts further toward positive bias, phase mismatching occurs among the electron waves propagating through the channels, leading to destructive interference and the observed drop in conductance.

**Table 1:** Experimental versus theoretical relative molecular conductance (*G_r_*) and conductance ratio pre/post C_60_ intercalation in Cu/Graphene/SAMs/EGaIn junctions.

SAMs	*G_r_* (Exp.)	R (Exp.)	Std (Exp.)	*G_r_* (Theo.)
**1**	8.4E‐5	2.3	1E‐5	1.2E‐5
**2**	2.0E‐4		6E‐5	1.3E‐4
**3**	3.6E‐5	8.3	1.E‐5	1.0E‐5
**4**	3.0E‐4		4.E‐5	5.0E‐4

SAM **4** exhibits a small degree of asymmetry in its *G–V* behavior, characterized by a curve transition at ≈−0.15 V. Nevertheless, this asymmetry is markedly less pronounced than that observed in SAM **2**. While DFT calculations predicted a greater degree of asymmetry, we attribute the subdued experimental asymmetry to the capture of more fullerene molecules within the SAM **4** cage. Interactions between the fullerene molecular systems likely broaden the resonance peak, suppressing the expected asymmetric transport behavior, while DFT calculations were operated on an idealized single molecular junction, where this interaction has not been considered. Measurements of TPT SAMs before and after fullerene immersion showed no significant shift in conductance, aligning with QCM results that indicated almost no fullerene incorporation within this densely packed monolayer (Figure S2, Supporting Information).

The Seebeck coefficient (S) is a critical parameter for molecular junctions, as it quantifies both the junction's potential utility in energy harvesting and the relative energetic position of the electrode Fermi level with respect to molecular frontier orbitals. Experimentally, S was determined by establishing a temperature difference (ΔT) across the SAMs. This was achieved by heating the copper substrate with a Peltier stage. A thermocouple monitored ΔT, while a differential preamplifier amplified the thermovoltage (V_Therm_) generated across the junction. The amplified V_Therm_ at varying ΔT values was recorded by a data acquisition card. Figure 2c,d illustrate the statistical distribution of V_Therm_ versus ΔT for SAMs **1–4**. A linear correlation between V_Therm_ and ΔT was observed in all cases. The negative slope obtained from linear regression corresponds to the Seebeck coefficient, *S = – V_Therm_/ΔT*. Table 2 lists the measured S values for all SAMs. Since S is independent of junction area, the absolute values are reported to facilitate comparison with other molecular junction systems. **Table** **2** summarizes S values obtained from both experimental measurements and theoretical calculations. All studied SAMs exhibit a positive *S*, indicating that the electrode Fermi level resides closer to the HOMO. An enhancement of *S* occurs upon introduction of the ZnTPP cap, as evident when comparing SAM **1** to SAM **3**, and SAM **2** to SAM **4**, in agreement with theoretical predictions. Transmission curve calculations indicate a shift of the electrode Fermi level toward the HOMO following cap addition. This S enhancement is explained by the higher slope in the transmission function near the molecular resonance (HOMO/LUMO) region, as S is determined from Mott's formula:

(2) $$S\;\approx -\frac{\pi^{2}k_{B}^{2}T}{3\left|e\right|}\left(\frac{\mathrm{dln}\left(T\left(E_{F}\right)\right)}{dE_{F}}\right)$$

**Table 2:** Experimental measured and theoretically calculated Seebeck and relative power factor (*PF_r_ = PF_SAMs_/PF_Graphene_*) for Cu/Graphene/SAMs/EGaIn junction.

SAMs	*S* [µV K^−1^]	STD	*S* [µV K^−1^]	*PF_r_*
	(Exp.)	[µV K^−1^]	(Theo.)	(Exp.)
**1**	51.0	9.2	74.5	3.2
**2**	49.0	7.7	69.8	7.1
**3**	56.3	4.1	79.5	1.7
**4**	55.0	8.5	78.3	14.0

A sharper transition yields a higher magnitude of (dlnT(E)/dE) at E_F_, resulting in an increased |S|. Comparison of SAMs before and after fullerene intercalation reveals nearly identical |S| values.

This indicates that the electrode Fermi level is not located exactly within the dip region, but rather within the flatter transmission plateau near the resonance part (Figure 4; Figure S3, Supporting Information).

### 2.3 Theoretical Understanding

The transport properties of the studied junctions were modelled using a combination of density functional theory (DFT) and quantum transport theory. To calculate electrical transport through junctions **1–4**, we modelled junctions formed from the single molecules shown in Figures S4 and S5 (Supporting Information) (for more details, see Optimised DFT Structures in the Supporting Information). Moreover, we calculated the frontier molecular orbitals for each multilayer, namely the highest occupied molecular orbital (HOMO), lowest unoccupied orbital (LUMO), and their neighbours (i.e., HOMO‐1, LUMO+1, etc.), along with their energies, as shown in Figures S6–S9 (Supporting Information). Since we are investigating multiple molecules with two different electrode materials and since the EGaIn surface is poorly characterized at an atomic scale, we modelled junctions formed from graphene and gold electrodes. The use of gold, rather than EGaIn, is in part supported by the fact that they have similar Fermi energies, because the work function of EGaIn was ≈4.3 eV, which is close to the gold work function (4.8 eV).^[^ ^20^ ^]^ To determine the optimum separation distance between the molecules and these electrodes, Figures S10–S15 (Supporting Information), shows the binding energies between two segments or electrodes and molecules as a function of their separation. The optimum distances between the two components correspond to the energy minima of these curves and are summarised in Table S1 (Supporting Information), (for more details, see Interfacial coupling strengths in the Supporting Information).

To compute the electrical conductance of multilayers **1–4**, we used the quantum transport code Gollum to obtain the transmission coefficient *T*(*E*) describing electrons of energy *E* passing from the source to the drain electrodes, from which the room‐temperature electrical conductance and Seebeck coefficient are determined as described in Equation S4 (Supporting Information). Figure S16 (Supporting Information) shows that after structural relaxation, when **1–4** multicomponent structures are placed between graphene and gold (Gr and Au), electrodes, they adopt an orthogonal orientation in which the pyridyl anchor faces the Au electrode and the large anchor (i.e., ZnTTP), faces the Gr electrode for **1–2** (see the Optimised DFT Structures of compounds in their junctions, SI). It has recently been demonstrated, by comparing *T*(*E*) for a single molecule against that of SAMs consisting of 7 molecules, that the *T*(*E*) for a SAM is approximately the same as for the single molecule. Figures S17 and S18 (Supporting Information) show the computed electrical conductances for multilayers **1–2**, this shows that **2** possesses a dip near the middle of the HOMO‐LUMO gap (near *E_F_ − E_F_^DFT^* = +0.2 eV), However, the conductance of **2** is higher than that of **1**. Similarly, the conductance of **3** is higher than that of **4**, as shown in Figures S19 and S20 (Supporting Information). This indicates that having C_60_ in the multilayer increases the conductance, as demonstrated in Figure 3a below. For example, the conductance of the orange curve is higher than the grey, and similarly the green curve is higher than the blue, such that (*G*_multicomponent_ < *G*_multicomponent+C60_), as shown in Figure 4 a. It should be noted that the C_60_ within the multilayer acts like a pendent (side) group, leading to a destructive quantum interference (DQI) feature within the gap (near $E_{F}-\;E_{F}^{DFT}$= +0.2 eV). Our DFT simulations reveal that electron transport through these multilayers typically takes place near the middle of the gap^[^ ^12^ ^]^ and indeed, we find that the closest agreement between theory and experiment is obtained at the DFT‐predicted Fermi level (where $E-\;E_{F}^{DFT}=0$), as shown in Table 1 below. Figure 3b, shows the corresponding Seebeck coefficients as a function of the Fermi energy *E_F_*. Since the sign of the Seebeck coefficient is determined by the slope of the transmission coefficient near the Fermi energy, and all multilayers are HOMO‐dominated, this causes the sign of the Seebeck coefficient to be positive. Furthermore, our DFT thermopower simulations suggest that the Seebeck coefficient for the studied multilayers should be approximately the same.

## 3 Conclusion

This study demonstrates a component‐to‐component strategy to decouple and synergistically optimize electrical conductivity and thermopower in self‐assembled monolayers (SAMs), addressing the fundamental limitation in conventional thermoelectric systems. The key advance on the supramolecular cavities had been reported previously^[^ ^20^ ^]^ is that the new architecture reported here enables controlled intercalation of fullerene derivatives within the SAM matrix, establishing an optimized material platform for high‐performance thermoelectric energy conversion systems. By engineering vertically aligned bipyridine frameworks on ZnTPP‐graphene substrates, we created ordered cavities for controlled fullerene intercalation, with architecture quality confirmed via QCM and AFM investigation. Electrical and thermoelectric measurements using the EGaIn system revealed that C_60_‐intercalated junctions exhibit a significant increase in normalized conductance compared to non‐intercalated analogs without a significant shift in Seebeck coefficient, resulting in enhanced power factor. Theoretical calculation attributes this enhancement to fullerene‐induced Fano resonance near the Fermi level. This work demonstrates a method of effectively intercalating molecules into a SAMs system and successfully achieves thermopower parameter decoupling via this intercalation, which provides a robust platform for high‐power‐factor molecular thermoelectrics, with implications for energy‐harvesting and tunable quantum behaviors.

## 4 Experimental Section

### SAMs Fabrication

ZnTPP SAMs and SAMs **1** were fabricated according to the previous work (ref. [20]).

ZnTPP SAMs preparation: 10mM Zinc Tetraphenylporphyrin (ZnTPP) solution was prepared by dissolving the molecule (>98%, Aladdin) into Dimethylformamide (DMF, >99.8%, Aladdin) solvent. Single layered graphene on copper (Six‐Carbon technology, quality characterized by Raman spectroscopy) were merged in solution for 20 min, rinsed with DMF (3 times), dichloromethane (DCM >99.9%, Aladdin) and ethanol (>99.5%, Sigma–Aldrich), and dried with nitrogen blow followed with incubating in nitrogen oven at 40 °C overnight.

SAMs **1** preparation: 10mM 4,4′‐biphenyl‐4,4′‐diyldipyridine molecules were prepared by dissolving the molecule (>95%, Aladdin) in DMF. Prepared ZnTPP SAMs were merged into solution for 12 h for the pyridine molecule to coordinate with the zinc center. SAMs after molecular backbone coordination growth were rinsed with DMF (3 times), dichloromethane, and ethanol, and dried with nitrogen blow followed with incubating in a nitrogen oven at 40 °C overnight.

SAMs **2** preparation: Prepared SAMs **1** were immersed in 0.1mg mL^−1^ fullerene solution (in toluene) for 24 h for fullerene doping. The doped SAMs were rinsed with toluene (3 times), dichloromethane, and ethanol, and dried with nitrogen blow followed with incubating in a nitrogen oven at 40 °C overnight.

SAMs **3** preparation: Prepared SAMs **1** was immersed in 10mM ZnTPP solution (in DMF) for 20 min, rinsed with DMF (3 times), dichloromethane, and ethanol, and dried with nitrogen blow followed with incubating in a nitrogen oven at 40 °C overnight.

SAMs **4** preparation: Prepared SAMs **1** were immersed in 0.1mg mL^−1^ fullerene solution (in toluene) for fullerene doping. After 24 h of immersion, ZnTPP molecules were gradually added to the fullerene solution until the concentration reached 10mM, waiting for 20 min for ZnTPP to seal the cage. Samples after SAMs growth were rinsed with toluene (3 times), dichloromethane and ethanol, and dried with nitrogen blow followed with incubating in a nitrogen oven at 40 °C overnight.

### SAMs Characterization

Growing step characterization: a series of analogous experiments was operated on a commercially purchased quartz nano‐crystal microbalance (QCM) Au substrate (5 mm diameter, initial frequency *f_0_* = 10 MHz, q‐Sensor). The crystal was rinsed with toluene, dichloromethane, and ethanol for several times, followed with incubation at high temperature (200 °C) for 30 min in a vacuum box. Graphene was transferred onto QCM substrate via standard recipe: Single‐layered graphene on copper (Six Carbon Technology, Graphene on 1 side, characterized by Raman) was spin coated with 4% poly (methyl methacrylate) (PMMA, 6000 rpm, 1 min), and placed on 0.1M FeCl_3_ solution for 24 h to etch‐off copper substrate, after this process, the PMMA protected graphene was floating on etching solvent surface. The graphene was fished by the annealed QCM crystal, washing off PMMA protection layer by immersing in 50 °C acetone for 1 h, and rinsing with acetone for several times. The graphene‐coated crystal was installed to a commercial QCM setup (OpenQCM), with frequency recorded as initial frequency (*f_0_′*).

SAMs ZnTPP and **1–4** were fabricated onto graphene‐coated crystal via the procedure explained in the SAMs fabrication procedure. The corresponding QCM frequency was recorded (f), and the frequency shift were calculated via the equation: *Δf = f_0_′ – f*. The mass of molecule (m) adsorbed onto the substrate were calculated via the Sauerbrey equation:

(3)

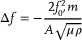

where *A* is the crystal area, *μ* is the quartz shear modulus, and *ρ* is the quartz density. The surface coverage was calculated via equation:

(4)

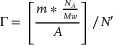

where *N’* is the estimated amount of molecules/area with 100% coverage, the molecular occupation area of ZnTPP was assumed to be ≈1.6 nm^[^ ^2^ ^]^ according to the previous work.

The *Γr* for SAM ZnTPP is obtained from QCM frequency shift (*Δf*) between graphene and ZnTPP SAMs. *Γr* for SAM **1** is obtained from *Δf* between SAM ZnTPP and SAM **1**. *Γr* for SAM 2 is obtained from *Δf* between SAM **1** and SAM **2**. *Γr* for SAM **3** is obtained from *Δf* between SAM **1** and SAM **3**. *Γr* for SAM **4** is obtained from *Δf* between SAM **1** and SAM **4**. The *Γ_r_* for all measured samples were listed in Table S3 (Supporting Information).

SAM's height characterization: Topology for SAMs **1–4** and ZnTPP were characterized by atomic force microscopy (AFM, a Bruker Multi‐Mode 8) in tapping mode, using a Multi‐75G (Budget Sensor, k = 3 N/m) probe, the integral gain and proportional gain were regulated dynamically during image scanning. The film thickness of prepared SAMs were obtained from the topological image obtained from the previous section. The height data topography image at the covered (*d_total_*) and uncovered (*d_Crystal_*) region was recorded via xyz file of AFM image, and the film thickness, *d_SAMs_*, were obtained via: *d_SAMs_ = d_Total_ – d_Crystal_*.

### Electric and Thermoelectric Characterization

EGaIn tip preparation: EGaIn tip preparation were according to the method published by Ryan et al. Eutectic alloy of gallium and indium (>99.99%, Sigma–Aldrich) in 10 µL syringe were used for tip preparation. A drop of liquid metal was extruded from the syringe tip (300 µm) and brought into contact with CuO substrate. The cone shapted EGaIn tip with 0.7 nm thick GaOx layer were prepared by slowly retracting the syringe. A micromanipulator (Narishige) were used to control the movement of the tip, and an elevating stage (PDV) was used to control the movement of the sample. A 1600x microscope (Leyue Z04‐1) was used to monitor the tip formation procedure.

Electric and thermoelectric measurements were performed using an EGaIn configuration, schematically illustrated in Figure S2 (Supporting Information). Temperature control was achieved via a Peltier stage powered by a DC source (DINGCE DC152D). A copper foil was positioned on the Peltier stage, electrically connected to a large metal plate serving as a ground shield. Electrical isolation was provided by a 25 µm mica sheet placed atop the copper foil. Subsequently, the self‐assembled monolayer (SAM) samples were placed onto the mica sheet. A localized region of the sample was subsequently ablated using a surgical blade to remove both the graphene layer and SAM coating, thereby exposing the underlying copper substrate. Two EGaIn tips, mounted on a micromanipulator, were incrementally advanced toward the sample surface until stable electrical contact was established: one tip contacting the SAM‐functionalized region and the other contacting the exposed copper area. The contact area between each EGaIn tip and the sample was quantified using optical microscopy. The contact area with the exposed copper was intentionally maximized to minimize contact resistance contributions.

During electrical characterization, the Peltier stage was disconnected from its power source. A Keithley 2400 SourceMeter was employed to characterize the junction's electrical behavior. Bias voltage was systematically swept between the two EGaIn tips. The resulting current was amplified using a low‐noise current preamplifier (Stanford Research Systems SR 570) and recorded by a data acquisition module (National Instruments NI‐USB‐6295). To prevent surface contamination of the EGaIn tips by ambient pollutants and ensure reproducible contact conditions, a fresh tip pair was prepared for each junction investigated. For each SAM system, a minimum of 120 current density‐voltage (*J–V*) profiles were acquired across ten or more distinct sample locations. Molecular conductance values (G) were extracted by performing numerical differentiation on the *J–V* data. Linear regression was applied over contiguous 5‐point segments (corresponding to a 10 mV window), with the calculated slope equated to the conductance at the central bias voltage.

Thermoelectric characterization was initiated by activating the Peltier stage via its power source. A thermocouple was affixed to the exposed copper region on the substrate to monitor the sample temperature (*T_Sample_*). A differential voltage preamplifier (Stanford Research Systems SR550) operating in A‐B mode was utilized to detect the thermoelectromotive force. Input ports A and B were connected to the EGaIn electrodes contacting the SAM region and the exposed copper region, respectively, enabling determination of the thermally induced voltage, ΔV. The temperature of the EGaIn probe contacting the SAM (*T_Probe_*) was assumed to approximate ambient temperature.

### Theoretical Simulations Section—DFT and Transport Calculations

The thermoelectric properties of the four junctions were further investigated using a combination of density functional theory and quantum transport theory to obtain the transmission coefficient *T*(*E*) describing electrons of energy *E* passing from the source to the drain electrodes. From this, the room‐temperature electrical conductance *G* and Seebeck coefficient *S* were determined using Equation S4 and Figures S17–S22 (Supporting Information), of the ESI. Figures S10–S15 (Supporting Information) demonstrate that a pyridyl‐terminated anthracene binds strongly to porphyrin (Zn‐TPP) and are shown in Table S2 (Supporting Information).

### Theoretical Simulations Section—Optimized DFT Structures

Geometries, electronic structures, and transport properties of all junctions were presented below. The main aim was to examine the change in transport properties of molecule/ZnTPP multilayers when they were inserted in Gr‐Au junctions for four different multilayers. Using the density functional code SIESTA, the optimum geometries of the isolated molecules were obtained by relaxing the molecules until all forces on the atoms were less than 0.01 eV/Å. A double‐zeta plus polarization orbital basis set, norm‐conserving pseudopotentials, an energy cut‐off of 250 Rydbergs defining the real space grid were used, and the local density approximation (GGA), was chosen as the exchange correlation functional. The basic building blocks I‐III and Zinc Tetraphenyl Porphyrin (ZnTPP, molecule III), combine with I and II to form multilayers of this study, were shown in Figure S4 (Supporting Information).

## Conflict of Interest

The authors declare no conflict of interest.

## Supporting information



Supporting Information

## Data Availability

The data that support the findings of this study are available from the corresponding author upon reasonable request.

## References

[1] D. Xiang , X. Wang , C. Jia , T. Lee , X. Guo , Chem. Rev. 2016, 116, 4318. 26979510 10.1021/acs.chemrev.5b00680

[2] M. Yuan , Y. Qiu , H. Gao , J. Feng , L. Jiang , Y. Wu , J. Am. Chem. Soc. 2024, 146, 7885. 38483827 10.1021/jacs.3c14044

[3] X. Huang , T. Li , J. Mater. Chem. C 2020, 8, 821.

[4] C. Yan , C. Fang , J. Gan , J. Wang , X. Zhao , X. Wang , J. Li , Y. Zhang , H. Liu , X. Li , J. Bai , J. Liu , W. Hong , ACS Nano 2024, 18, 28531. 39395180 10.1021/acsnano.4c10389

[5] K. Yase , Jpn. J. Appl. Phys. 2024, 63, 120804.

[6] H. Fruechtl , T. van Mourik , Phys. Chem. Chem. Phys. 2021, 23, 1811. 33443268 10.1039/d0cp06250b

[7] Y. Kim , W. Jeong , K. Kim , W. Lee , P. Reddy , Nat. Nanotechnol. 2014, 9, 881. 25282046 10.1038/nnano.2014.209

[8] X. Wang , A. Ismael , A. Almutlg , M. Alshammari , A. Al‐Jobory , A. Alshehab , T. L. Bennett , L. A. Wilkinson , L. F. Cohen , N. J. Long , Chem. Sci. 2021, 12, 5230. 34163759 10.1039/d1sc00672j PMC8179551

[9] P. He , J. Jang , H. Kang , H. J. Yoon , Chem. Rev. 2025, 125, 2953. 39908450 10.1021/acs.chemrev.4c00886

[10] L. Cui , R. Miao , K. Wang , D. Thompson , L. Angela Zotti , J. Carlos Cuevas , E. Meyhofer , P. Reddy , Nat. Nanotechnol. 2018, 13, 122. 29255291 10.1038/s41565-017-0020-z

[11] J. C. Kloeckner , R. Siebler , J. C. Cuevas , F. Pauly , Phys. Rev. B 2017, 95, 245404.

[12] L. Rincon‐Garcia , A. K. Ismael , C. Evangeli , I. Grace , G. Rubio‐Bollinger , K. Porfyrakis , N. Agrait , C. J. Lambert , Nat. Mater. 2016, 15, 289. 26641017 10.1038/nmat4487

[13] P. Reddy , S.‐Y. Jang , R. A. Segalman , A. Majumdar , Science 2007, 315, 1568. 17303718 10.1126/science.1137149

[14] A. K. Ismael , L. Rincón‐García , C. Evangeli , P. Dallas , T. Alotaibi , A. A. Al‐Jobory , G. Rubio‐Bollinger , K. Porfyrakis , N. Agraït , C. J. Lambert , Nanoscale Horiz. 2022, 7, 616. 35439804 10.1039/d1nh00527h

[15] S. V. Aradhya , L. Venkataraman , Nat. Nanotechnol. 2013, 8, 399. 23736215 10.1038/nnano.2013.91

[16] R. Miao , H. Xu , M. Skripnik , L. Cui , K. Wang , K. G. L. Pedersen , M. Leijnse , F. Pauly , K. Warnmark , E. Meyhofer , P. Reddy , H. Linke , Nano Lett. 2018, 18, 5666. 30084643 10.1021/acs.nanolett.8b02207

[17] X. Wang , T. L. R. Bennett , A. Ismael , L. A. Wilkinson , J. Hamill , A. J. P. White , I. M. Grace , O. V. Kolosov , T. Albrecht , B. J. Robinson , N. J. Long , L. F. Cohen , C. J. Lambert , J. Am. Chem. Soc. 2020, 142, 8555. 32343894 10.1021/jacs.9b13578 PMC7588028

[18] S. K. Lee , R. Yamada , H. Tada , Mater. Res. Innovations 2014, 18, S6.

[19] S. Park , H. R. Kim , J. Kim , B.‐H. Hong , H. J. Yoon , Adv. Mater. 2021, 33, 2103177. 10.1002/adma.202103177 34453364

[20] X. Wang , A. Ismael , B. Alanazi , A. Al‐Jobory , J. Wang , C. J. Lambert , J. Mater. Chem. C 2023, 11, 14652.

[21] T. L. Bennett , M. Alshammari , S. Au‐Yong , A. Almutlg , X. Wang , L. A. Wilkinson , T. Albrecht , S. P. Jarvis , L. F. Cohen , A. Ismael , Chem. Sci. 2022, 13, 5176. 35655580 10.1039/d2sc00078d PMC9093172

[22] S. Park , J. Jang , Y. Tanaka , H. J. Yoon , Nano Lett. 2022, 22, 9693. 36441166 10.1021/acs.nanolett.2c03974

[23] S. Park , H. J. Yoon , Nano Lett. 2018, 18, 7715. 30418032 10.1021/acs.nanolett.8b03404

[24] A. Ismael , X. Wang , T. L. R. Bennett , L. A. Wilkinson , B. J. Robinson , N. J. Long , L. F. Cohen , C. J. Lambert , Chem. Sci. 2020, 11, 6836. 33033599 10.1039/d0sc02193h PMC7504895

[25] H. B. Akkerman , P. W. M. Blom , D. M. de Leeuw , B. de Boer , Nature 2006, 441, 69. 16672966 10.1038/nature04699

[26] X. Wang , X. Li , S. Ning , A. Ismael , J. Mater. Chem. C 2023, 11, 12348.

[27] S. Park , J. Jang , H. Kim , D. I. Park , K. Kim , H. J. Yoon , J. Mater. Chem. A 2020, 8, 19746.

[28] X. Wang , A. Ismael , B. Alanazi , A. Al‐Jobory , J. Wang , C. J. Lambert , J. Mater. Chem. C 2023, 11, 14652.

[29] Y. Li , L. Xiang , J. L. Palma , Y. Asai , N. Tao , Nat. Commun. 2016, 7, 11294. 27079152 10.1038/ncomms11294 PMC4835548

[30] F. Pauly , J. K. Viljas , J. C. Cuevas , Phys. Rev. B 2008, 78, 035315.

[31] S. Park , S. Kang , H. J. Yoon , Nano Lett. 2022, 22, 3953. 35575639 10.1021/acs.nanolett.2c00422

[32] M. Alshammari , A. A. Al‐Jobory , T. Alotaibi , C. J. Lambert , A. Ismael , Nanoscale Adv. 2022, 4, 4635. 36341305 10.1039/d2na00515h PMC9595198

[33] P. Reddy , S. Y. Jang , R. A. Segalman , A. Majumdar , Science 2007, 315, 1568. 17303718 10.1126/science.1137149

[34] A. K. Ismael , I. Grace , C. J. Lambert , Nanoscale 2015, 7, 17338. 26426840 10.1039/c5nr04907e

[35] X. T. Wang , S. Sangtarash , A. Lamantia , H. Dekkiche , L. Forcieri , O. Kolosov , S. P. Jarvis , M. R. Bryce , C. J. Lambert , H. Sadeghi , B. J. Robinson , J. Phys. Energy 2022, 4, 024002.

[36] S. Yoshimoto , E. Tsutsumi , K. Suto , Y. Honda , K. Itaya , Chem. Phys. 2005, 319, 147.

[37] J. Ye , A. Al‐Jobory , Q.‐C. Zhang , W. Cao , A. Alshehab , K. Qu , T. Alotaibi , H. Chen , J. Liu , A. K. Ismael , Sci. China Chem. 2022, 65, 1822.

[38] J. Chen , T. J. Giroux , Y. Nguyen , A. A. Kadoma , B. S. Chang , B. VanVeller , M. M. Thuo , Phys. Chem. Chem. Phys. 2018, 20, 4864. 29384159 10.1039/c7cp07531f

[39] F. C. Simeone , H. J. Yoon , M. M. Thuo , J. R. Barber , B. Smith , G. M. Whitesides , J. Am. Chem. Soc. 2013, 135, 18131. 24187999 10.1021/ja408652h

