# Supplementary material for: Layer‐by‐Layer Fabrication of Fullerene‐Intercalated Orthogonal Molecular Architectures Enhances Thermoelectric Behavior of Graphene‐Based Nanodevices
Source: Small. 2025 Aug 29;21(42):e07988. doi: 10.1002/smll.202507988 (PMC12548007; doi:10.1002/smll.202507988)
Supplement: Supplementary file 1 — Supporting Information [file SMLL-21-e07988-s001.docx]

Supporting Information

Layer-by-Layer Fabrication of Fullerene-Intercalated Orthogonal Molecular Architectures Enhances Thermoelectric Behavior of Graphene-Based Nanodevices

Ali Ismael,^a*^ Xintai Wang,^b, c*^ Bashayr Alanazi,^a^ Alaa Al-Jobory,^a^ and Colin J. Lambert,^a*^

**Figure S1** Correlation between Γr for SAMs ZnTPP and Γr for SAMs **1, 2** and **4.**


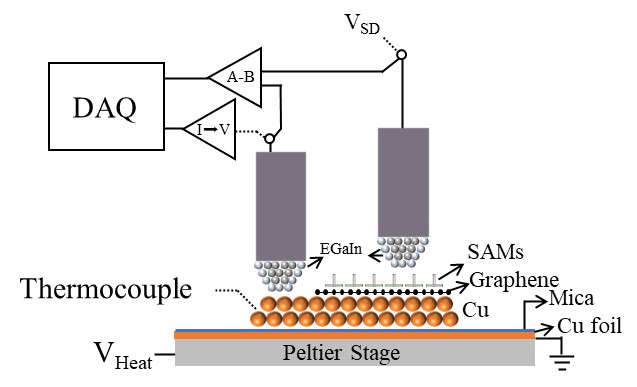


**Figure S2** Illustration of thermoelectric measurement system used in this work.


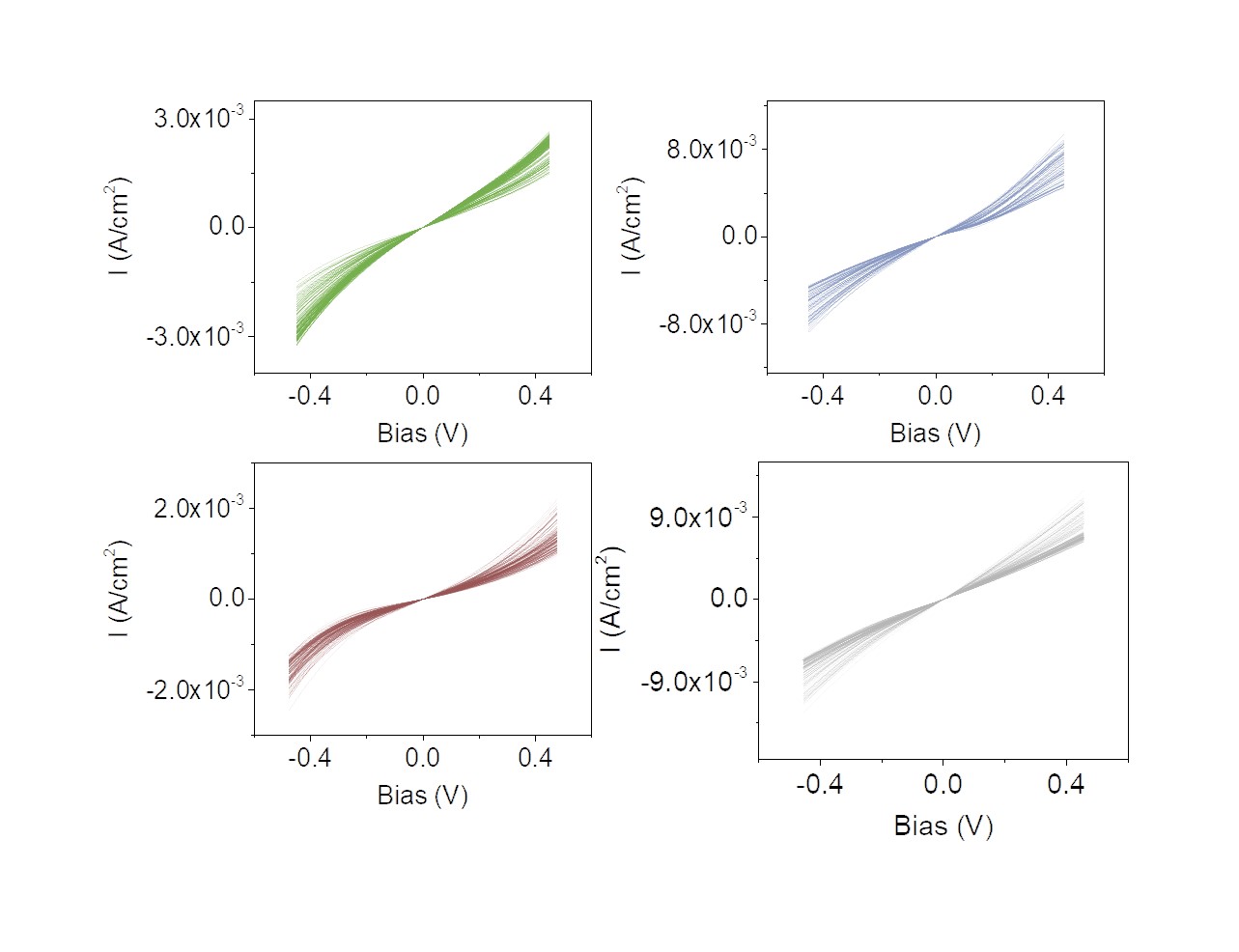


**Figure S1** Statistical IV curves for SAMs **1** (a), **2** (b), **3** (c) and **4** (d).


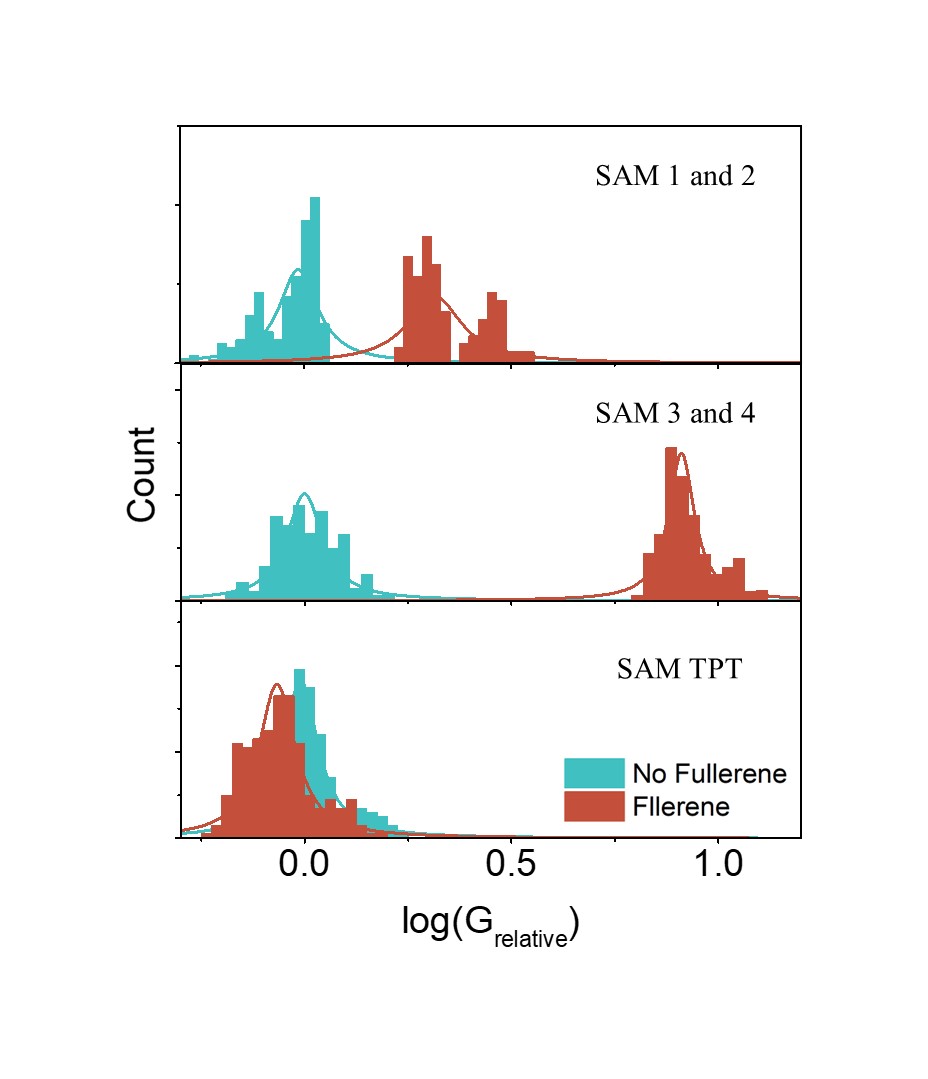


**Figure S2** Conductance distribution shift of SAM **1**, SAM **2** and SAM TPT before and after fullerene intercalation. G_relative_ refers to G_SAMs_/G_(No Fullerene SAMs, Avg)_


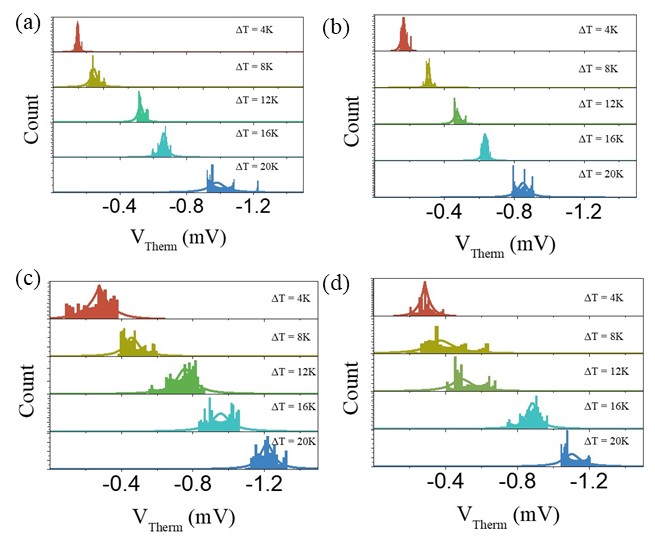


**Figure S3** (a-d) Plot of thermo-voltage (V_Therm_) vs. ΔT for SAMs **1-4**

Optimised DFT Structures of Isolated Molecular-scale Structures

Using the SIESTA1^1^ code, the optimum geometries of the isolated molecules were obtained by relaxing the molecules until all forces on the atoms were less than 0.01 eV / Å. A double-zeta plus polarization orbital basis set, norm-conserving pseudopotentials, an energy cut-off of 250 Rydbergs defining the real space grid were used and the local density approximation (GGA)^2-4^ was chosen as the exchange correlation functional. The basic building blocks I-III of this study are shown in Figure S4. Zinc Tetraphenyl Porphyrin (ZnTPP, molecule III), combines with I and II to form multilayers.

#
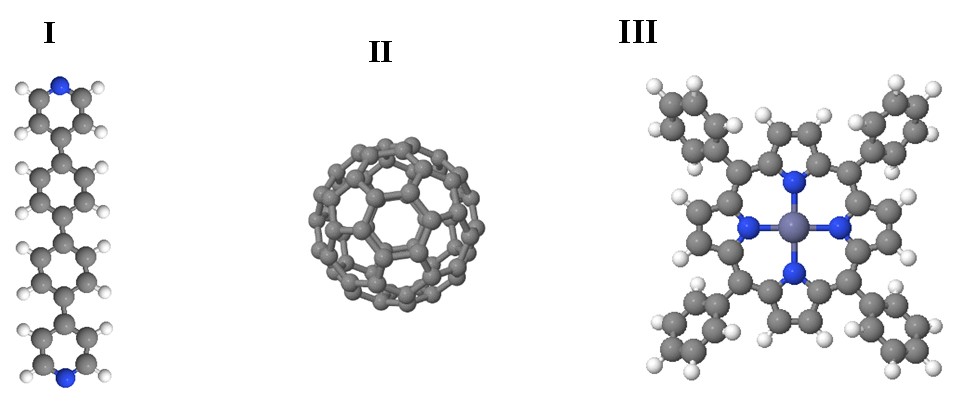


**Figure S4** DFT simulated structures of monolayers **I-III**.

The following 3 molecular structures were assembled by combining the ZnTPP with I and II (**Figure S5**), and then allowing the system to become fully relaxed to form multilayers (**1-4**), as shown in **Figure S5**.


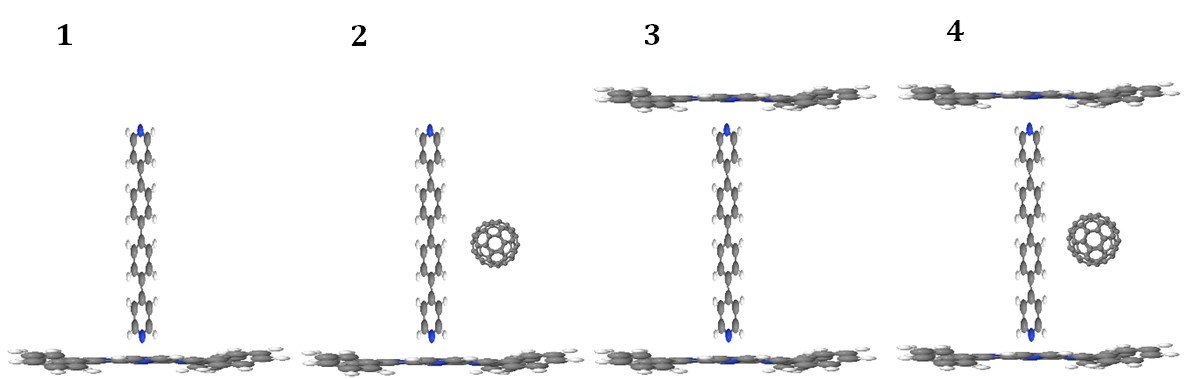


**Figure S5** DFT structure of SAMs **1-4.**

Frontier molecular orbitals

To understand the electronic properties of the structures of the studied molecules (see **Figures S5** and **S6**), the gas-phase electronic structures of all cross-linking were investigated to explore the distribution and composition of the frontier molecular orbitals. Plots of the frontier orbitals for the OPE molecule and multi-component molecules are given in **Figures S6-S9** the highest occupied molecular orbitals (HOMO) and lowest unoccupied orbitals (LUMO), (HOMO-1), and (LUMO+1), (HOMO-2), and (LUMO+2), (HOMO-3) and (LUMO+3) along with their energies. The blue and red colours represent the positive and negative orbital amplitude respectively.

**SAMs 1**


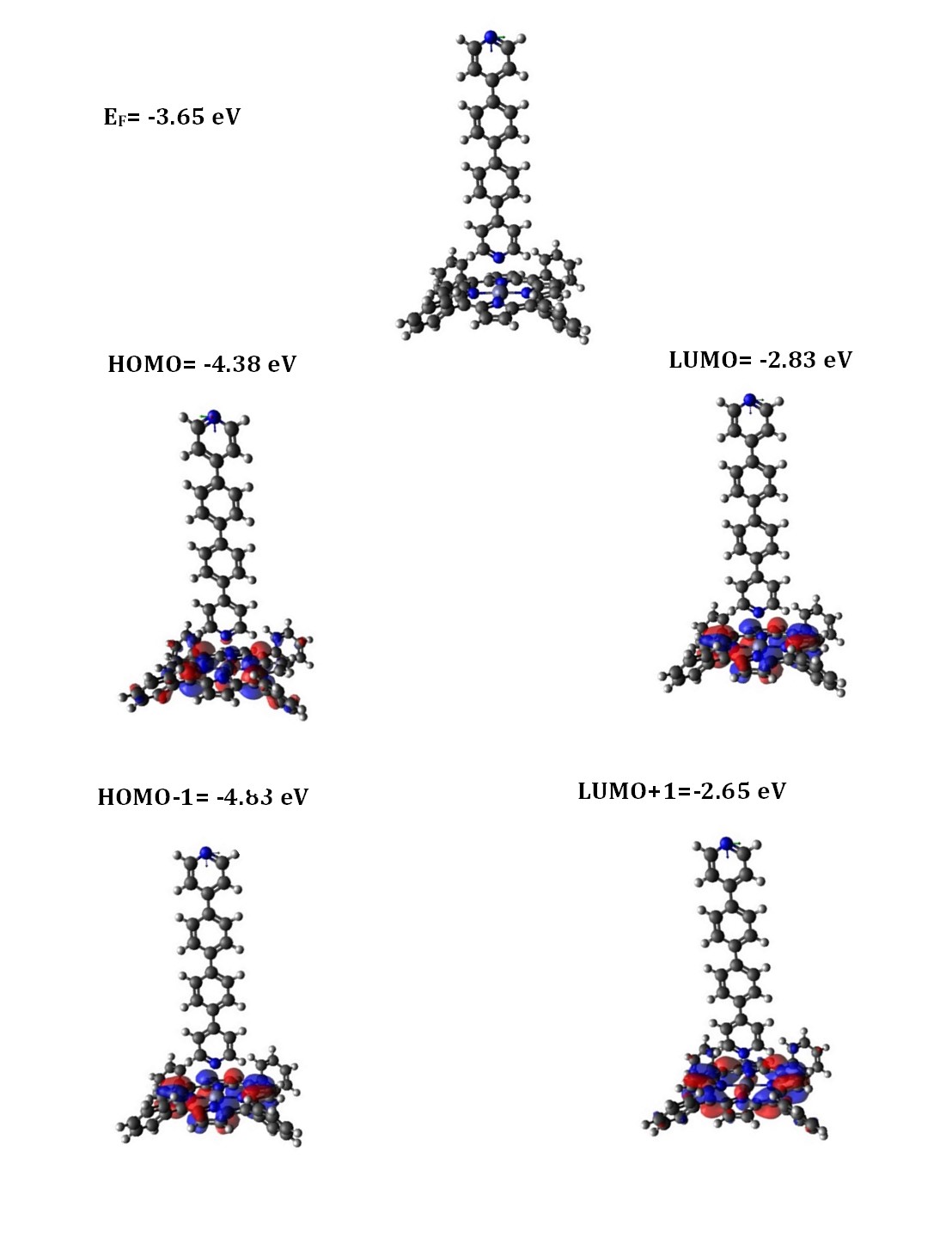


**Figure S6**: Wave function for multilayer **1**. Top panel: fully optimised geometry of multilayer 1 Lower panel: HOMO, LUMO, HOMO-1, LUMO+1, HOMO-2, LUMO+2, SAMs **1**, along with their energies.

**SAMs 2**


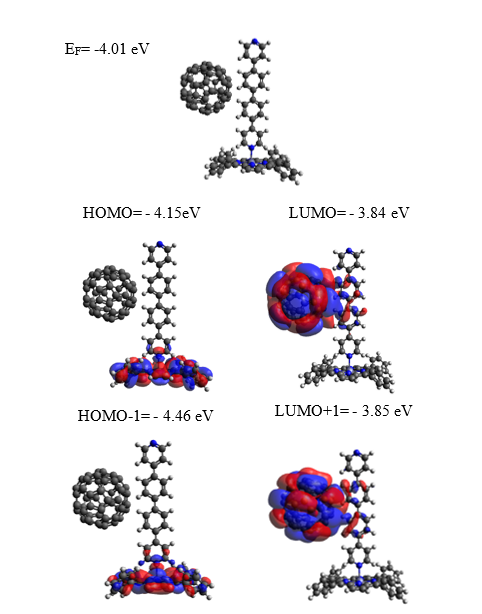


**Figure S7**: Wave function for multilayer **2**. Top panel: fully optimised geometry of multilayer **2**. Lower panel: HOMO, LUMO, HOMO-1, LUMO+1, HOMO-2, LUMO+2 of SAMs **2**, along with their energies.

**SAMs 3**


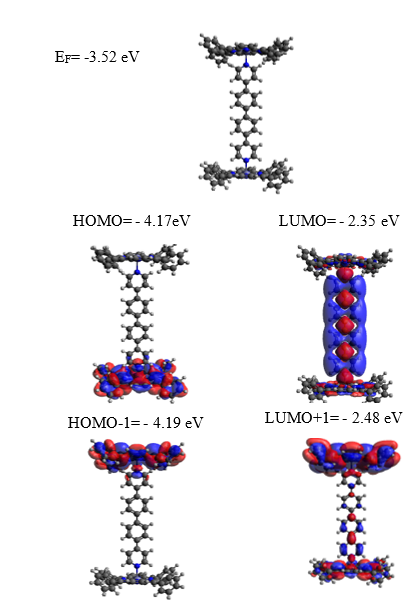


**Figure S8**: Wave function for multilayer **3**. Top panel: fully optimised geometry of multilayer **3**. Lower panel: HOMO, LUMO, HOMO-1, LUMO+1, HOMO-2, LUMO+2 of SAMs **3**, along with their energies.

**SAMs 4**

**
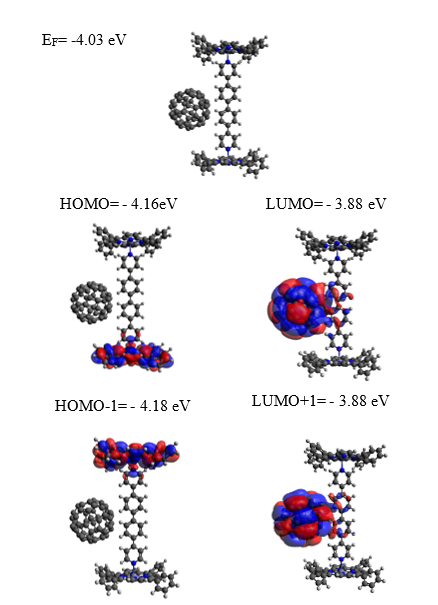
**

**Figure S9:** Wave function for multilayer **4**. Top panel: fully optimised geometry of multilayer **4**. Lower panel: HOMO, LUMO, HOMO-1, LUMO+1, HOMO-2, LUMO+2 of SAMs **4** along with their energies.

Interfacial coupling strengths:

To calculate the optimum binding distance between any two components, we used DFT and the counterpoise method, which removes basis set superposition errors (BSSE). The binding distance d is defined as the distance between compound 1 and compound 2. Here, compound 1 is defined as entity A and compound 2 as entity B. The ground state energy of the total system is calculated using SIESTA and is denoted$\mathbf{E}_{\mathbf{AB}}^{\mathbf{AB}}$. The energy of each entity is then calculated in a fixed basis, which is achieved using ghost atoms in SIESTA. Hence, the energy of the individual 1 in the presence of the fixed basis is defined as $\mathbf{E}_{\mathbf{A}}^{\mathbf{AB}}$ and for the gold as $\mathbf{E}_{\mathbf{B}}^{\mathbf{AB}}$. The binding energy is then calculated using the following equation^5-7^:

#

|  | $\mathbf{Binding Energy=}\mathbf{E}_{\mathbf{AB}}^{\mathbf{AB}}\mathbf{-}\mathbf{E}_{\mathbf{A}}^{\mathbf{AB}}\mathbf{-}\mathbf{E}_{\mathbf{B}}^{\mathbf{AB}}$ | (S1) |
| --- | --- | --- |

**Py/ZnTPP**

The optimum distance between the ZnTTP molecule and pyridyl anchor is found to be about 2.3 Å, at approximately -0.5 eV as shown in **Figure S10**.


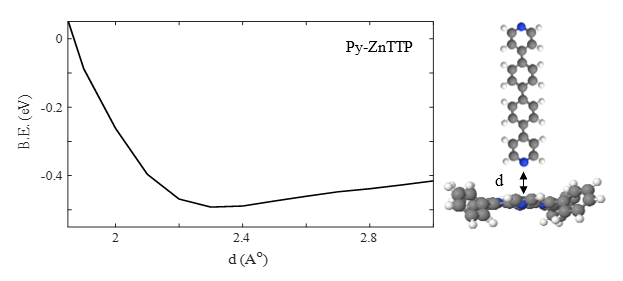


**Figure S10:** Right panel: represents the ZnTTP binding to molecule I through Zn atom. Left panel: Binding energy as a function of the optimum binding distance 𝑑, where 𝑑 is found to be approximately 2.3 Å, and binding energy B.E= 0.5 eV.

**Fullerene/backbone**

The optimum distance between the Py-ZnTTP and C_60_ is found to be about 3.4 Å, at approximately -0.2 eV as shown in **Figure S11**.


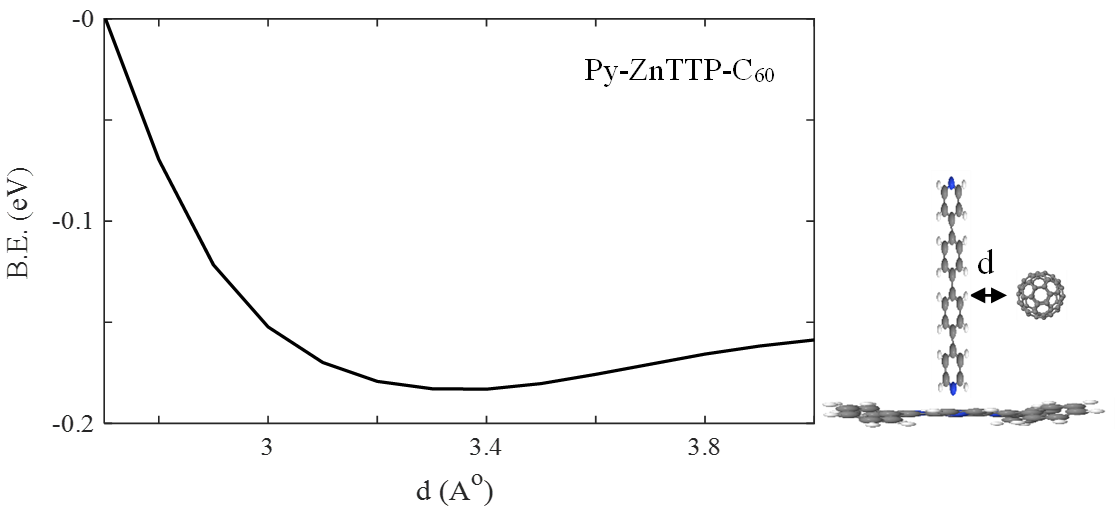


**Figure S11:** Right panel: represents molecule C_60_ binding to Py-ZNTTP. Left panel: Binding energy as a function of the optimum binding distance 𝑑, where 𝑑 is found to be approximately 3.4 Å, and binding energy B. E= 0.20 eV.

**Py/Au**

The optimum distance between the Au electrode and pyridyl anchor is found to be about 2.3 Å, at approximately -0.4 eV as shown in **Figure S12**.


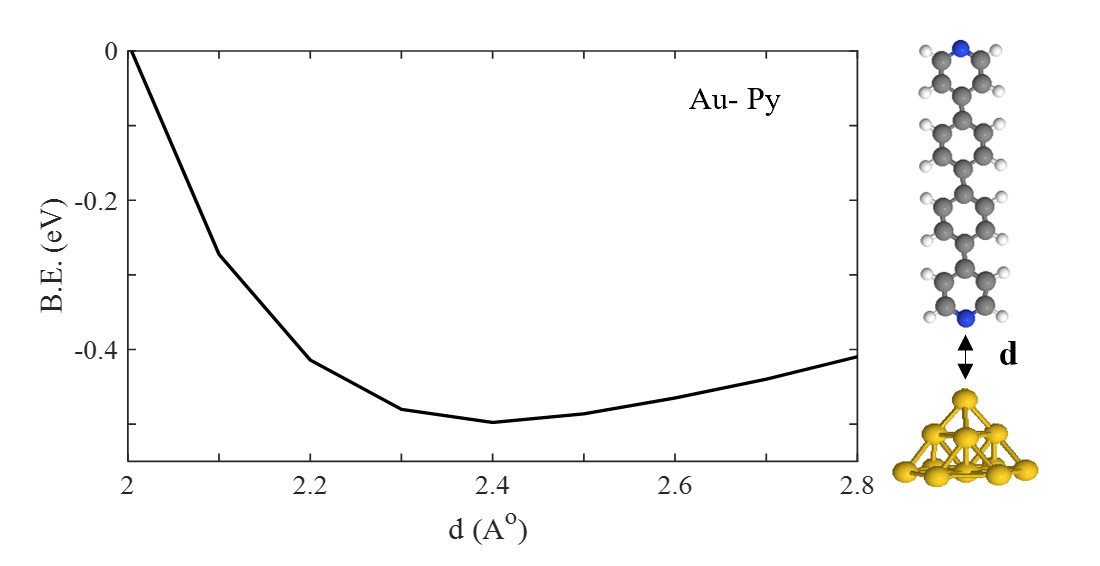


**Figure S12**: Right panel: represents molecule **I** binding to a gold ad-atom. Left panel: Binding energy as a function of the optimum binding distance 𝑑, where 𝑑 is found to be approximately 2.3 Å, and binding energy B.E= 0.4 eV.

**Au/ZnTTP**

The optimum distance between the Au electrode and Zn atom is found to be about 2.9 Å, at approximately -0.5 eV as shown in **Figure S13**.


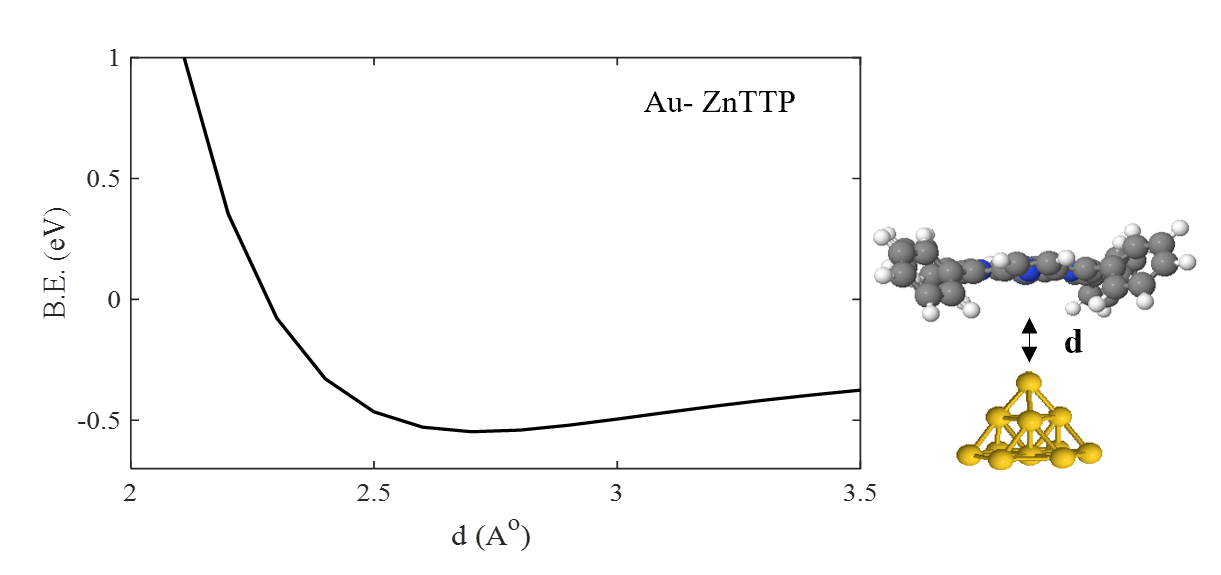


**Figure S13:** Right panel: represents ZnTTP molecule binding to gold ad-atom. Left panel: Binding energy as a function of the optimum binding distance 𝑑, where 𝑑 is found to be approximately 2.9 Å, and binding energy B.E= 0.5 eV.

**Graphene/ZnTTP**

The optimum distance between the Au electrode and pyridyl anchor is found to be about 4.0 Å, at approximately -0.2 eV as shown in **Figure S14**.


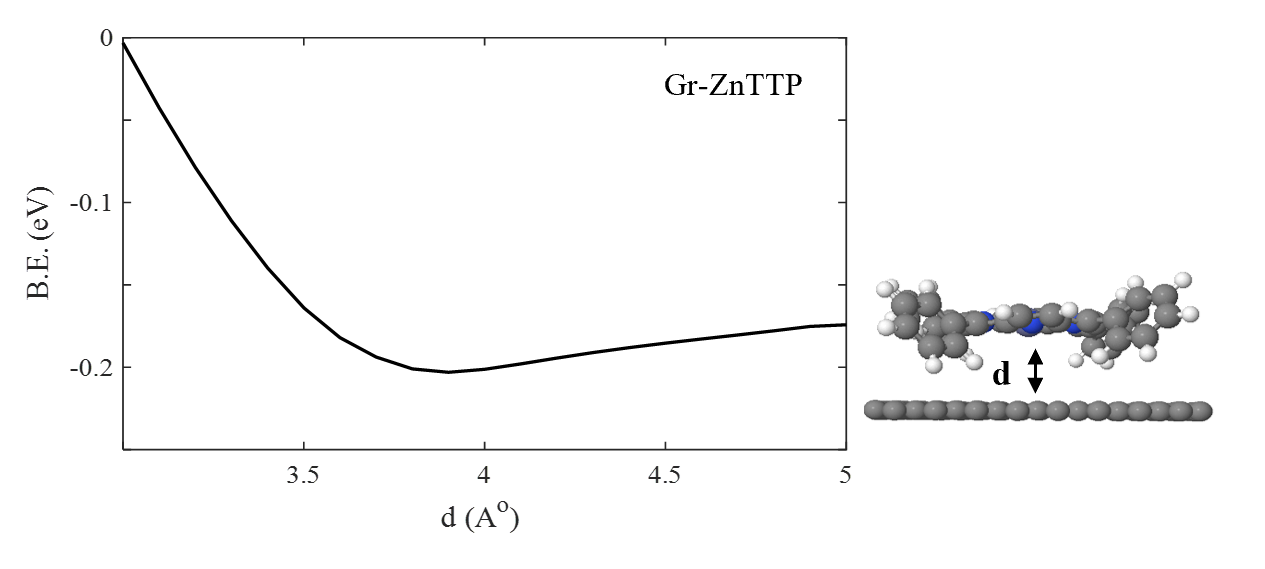


**Figure S14**: Right panel: represents the ZnTTP molecule binding to graphene sheet. Left panel: Binding energy as a function of the optimum binding distance 𝑑, where 𝑑 is found to be approximately 4.0 Å, and binding energy B.E= 0.2 eV.

**Graphene-Py-ZnTTP**

The optimum distance between bilayer and graphene sheet anchor is found to be about 4.0 Å, at approximately -0.15 eV as shown in **Figure S15**.


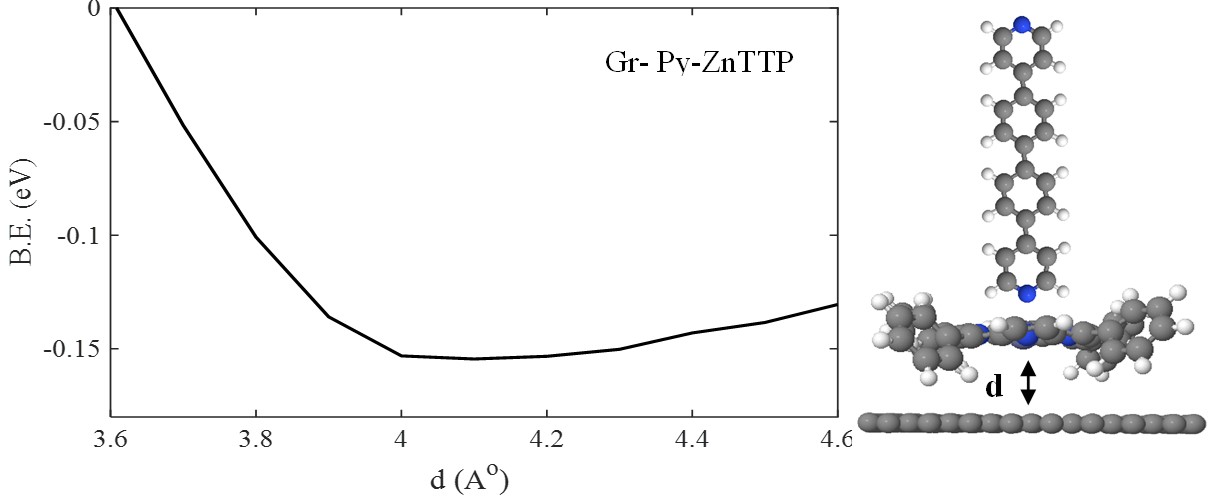


**Figure S15**: Right panel: represents bilayer Py-ZnTTP binding to graphene sheet. Left panel: Binding energy as a function of the optimum binding distance 𝑑, where 𝑑 is found to be approximately 4.0 Å, and binding energy B.E= 0.15 eV.

**Junction Structure DFT Optimization**

Using the optimized structures and geometries for the compounds obtained as described above, we again employed the SIESTA code to calculate self-consistent optimized geometries, ground state Hamiltonians and overlap matrix elements for each graphene-SAMs-gold junction. SAMs **1-4** sandwiched between gold and single layer graphene sheet (SLG).

The studied systems (**1-4**), are sandwiched between a single layer of graphene as a bottom electrode and gold as shown in **Figure S16**.


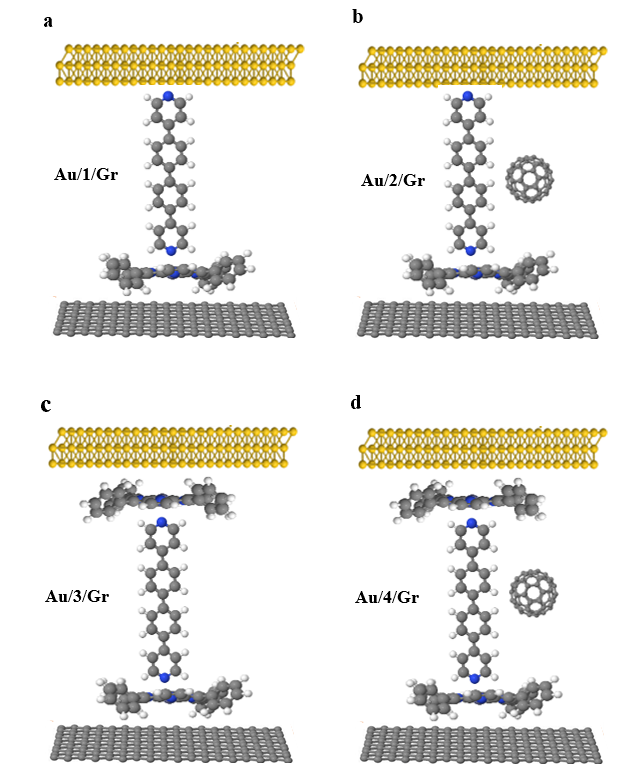


**Figure S16:** Schematic illustration of the Au/multilayer/SLG junctions. Top contact is Au electrode and the bottom contact is SLG electrode. a-d: Au/SAMs **1-4**/SLG.

**Transport Simulation in SLG-Au Junction**

The transmission coefficient T(E) is defined as the probability of transmission of electron with energy E that can pass through a molecule from one electrode to another^7^. The electrical conductance G/G_0_ were calculated using Gollum transport code for each junction Au/**1-4**/Gr, as shown in **Figures S17-S21** based on the binding energy simulations in **Table S1**. The Fermi level of the electrodes in all systems were 0 eV.


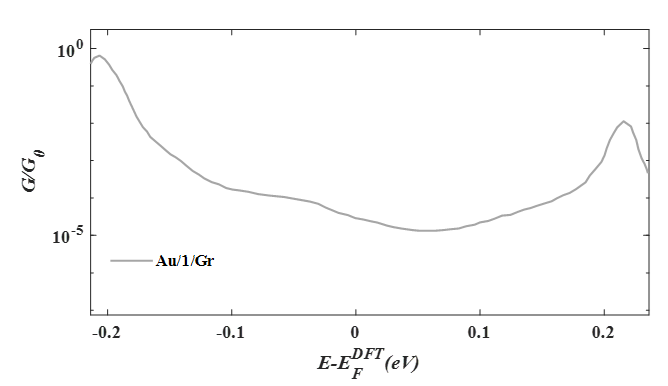


**Figure S17**: The calculated conductance as a function of the Fermi energy for Au/**1**/Gr junction.


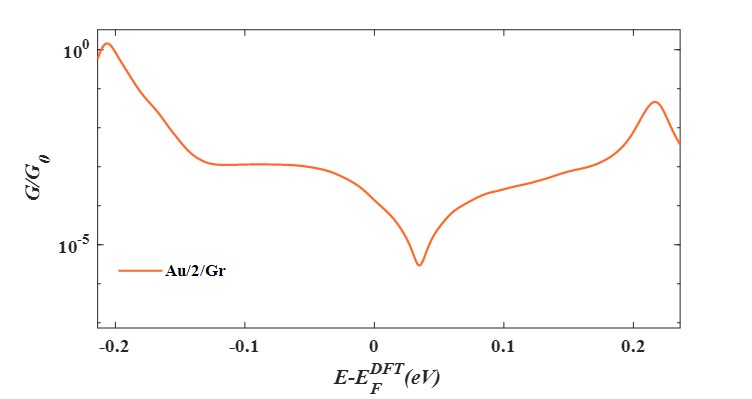


**Figure S18:** The calculated conductance as a function of the Fermi energy for Au/**2**/Gr junction.


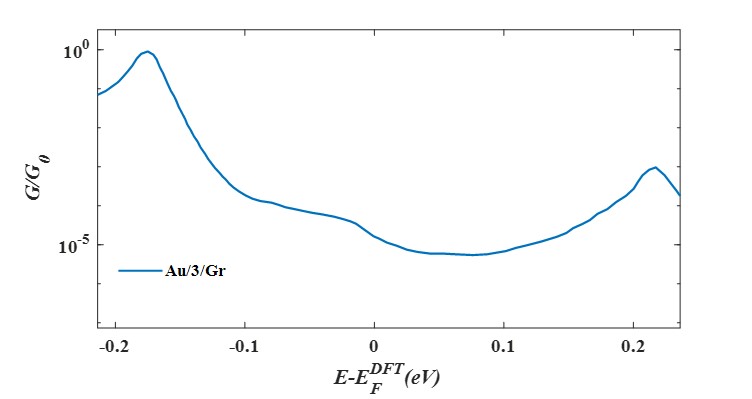


**Figure S19**: The calculated conductance as a function of the Fermi energy for Au/**3**/Gr junction


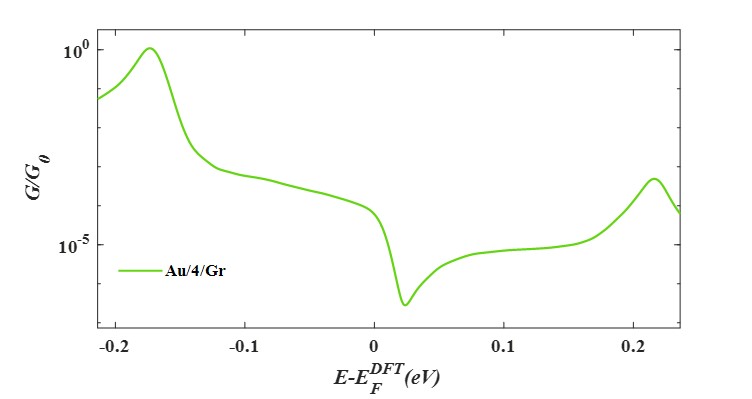


**Figure S20:** The calculated conductance as a function of the Fermi energy for Au/4/Gr junction

.
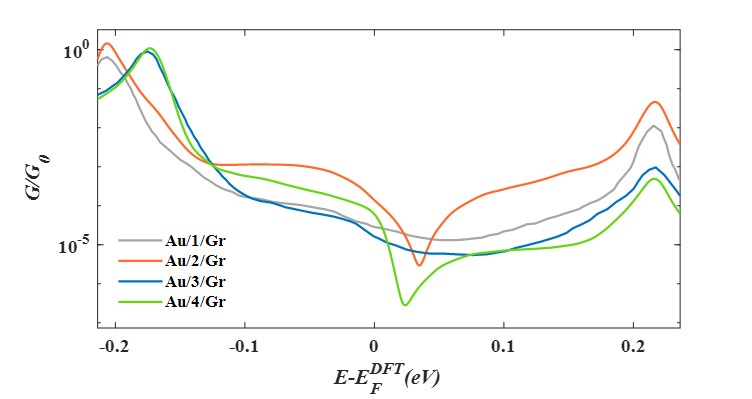


**Figure S21:** The calculated conductance as a function of the Fermi energy for Au/**1-4**/Gr junctions

**Thermopower Simulation**

To calculate the thermopower of the studied molecular junctions, it is useful to introduce the non-normalised probability distribution $P(E)$ defined by

|  | $P\left( E \right)=-T(E)\frac{df(E)}{dE}$ | (S2) |
| --- | --- | --- |

where $f(E)$ is the Fermi-Dirac function and $T(E)$ is the transmission coefficients and whose moments $L_{i}$ are denoted as follows

|  | $L_{i}=\int dEP(E){(E-E_{F})}^{i}$ | (S3) |
| --- | --- | --- |

where $E_{F}$ is the Fermi energy. The Seebeck coefficient, $S$, is then given by

|  | $S\left( T \right)=-\frac{1}{eT}\frac{L_{1}}{L_{0}}$ | (S4a) |
| --- | --- | --- |

where $e$ is the magnitude of the electronic charge. Similarly, the electrical conductance is given by

$\frac{G}{G_{0}}=L_{0}$ (S4b)

where $G_{0}$ is the quantum of conductance.


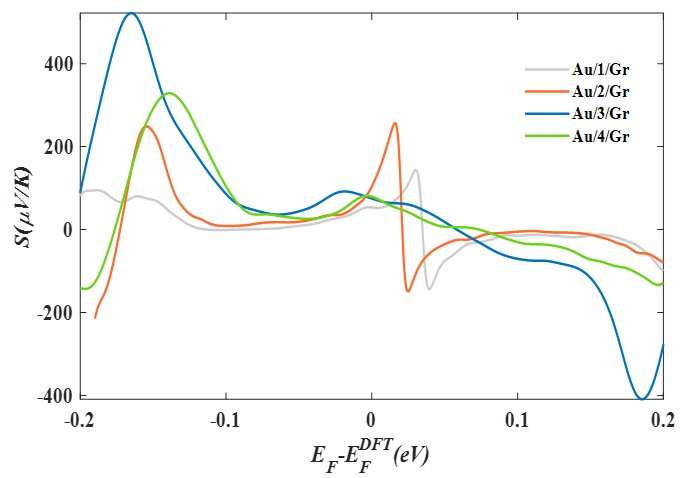


**Figure S22:** Seebeck coefficient S as a function of Fermi energy of the studied junctions. Seebeck coefficients of SAMs **1-4**, grey, orange, blue and green curves respectively.

**Table S1** Thermoelectric result in absolute value:

| **SAMs** | **G**  **(S/cm^2^)** | **std**  **(S/cm^2^)** | **S**  **(μV/K)** | **std**  **(μV/K)** | **PF**  **(μWK^-1^/ cm^2^)** |
| --- | --- | --- | --- | --- | --- |
| 1 | 0.46 | 0.05 | 51 | 9.2 | 0.36 |
| 2 | 1.1 | 0.3 | 49 | 7.7 | 0.78 |
| 3 | 0.2 | 0.06 | 56.3 | 4.1 | 0.19 |
| 4 | 1.65 | 0.2 | 55 | 8.5 | 1.51 |

**Table S2** Summarises all the binding energies (B.E), and optimum distances (𝑑), calculations for mono/bilayers that bind to gold or graphene sheet at different contact points.

| Contact point | 𝑑 (Å) | B.E (eV) |
| --- | --- | --- |
| Py-ZnTTP | 2.3 | 0.50 |
| Py-ZnTTP-C_60_ | 3.4 | 0.20 |
| Au-Py | 2.3 | 0.40 |
| Gr-ZnTTP | 4.0 | 0.20 |
| Gr-Py-ZnTTP | 4.0 | 0.15 |

**Table S3** Γ_r_ for all measured samples in this work.

| **Samples** | **ZnTPP**  **(%)** | **SAM 1**  **(%)** | **SAM2**  **(%)** | **SAM3**  **(%)** | **SAM4**  **(%)** |
| --- | --- | --- | --- | --- | --- |
| 1 | 122 | 91 | 55 | - | - |
| 2 | 145 | 77 | 49 | - | - |
| 3 | 131 | 95 | 67 | - | - |
| 4 | 133 | 93 | - | 105 | - |
| 5 | 109 | 88 | - | 113 | - |
| 6 | 135 | 81 | - | - | 84 |
| 7 | 151 | 82 | - | - | 91 |
| 8 | 145 | 91 | - | - | 78 |

# References

1. Soler, J. M.; Artacho, E.; Gale, J. D.; García, A.; Junquera, J.; Ordejón, P.; Sánchez-Portal, D. J. J. o. P. C. M., The SIESTA method for ab initio order-N materials simulation. *J. Phys.: Condens. Matter* 2002, *14* (11), 2745.

2. Perdew, J. P.; Burke, K.; Ernzerhof, M., Generalized gradient approximation made simple. *Phys. Rev. Lett.* 1996, *77* (18), 3865.

3. Becke, A. D., Density-functional exchange-energy approximation with correct asymptotic behavior. *Phys. Rev. A* 1988, *38* (6), 3098.

4. Perdew, J. P.; Wang, Y., Accurate and simple analytic representation of the electron-gas correlation energy. *Physical review B* 1992, *45* (23), 13244.

5. Kobko, N.; Dannenberg, J., Effect of basis set superposition error (BSSE) upon ab initio calculations of organic transition states. *The Journal of Physical Chemistry A* 2001, *105* (10), 1944-1950.

6. Sherrill, C. D., Counterpoise correction and basis set superposition error. *School of Chemistry and Biochemistry, Georgia Institute of Technology* 2010.

7. Sinnokrot, M. O.; Valeev, E. F.; Sherrill, C. D., Estimates of the ab initio limit for π− π interactions: The benzene dimer. *Journal of the American Chemical Society* 2002, *124* (36), 10887-10893.
